# Removal of H2Aub1 by ubiquitin-specific proteases 12 and 13 is required for stable Polycomb-mediated gene repression in *Arabidopsis*

**DOI:** 10.1186/s13059-020-02062-8

**Published:** 2020-06-16

**Authors:** Lejon E. M. Kralemann, Shujing Liu, Minerva S. Trejo-Arellano, Rafael Muñoz-Viana, Claudia Köhler, Lars Hennig

**Affiliations:** 1 Department of Plant Biology, Uppsala BioCenter, Swedish University of Agricultural Sciences and Linnean Centre for Plant Biology, SE-75007 Uppsala, Sweden; 2 Instituto de Neurociencias, Universidad Miguel Hernández-Consejo Superior de Investigaciones Científicas, 03550 Sant Joan d’Alacant, Spain

**Keywords:** H2AK121ub, H2Aub, H3K27me3, PRC1, PRC2, EMF1, LHP1, REF6, DUB, Responsiveness

## Abstract

**Background:**

Stable gene repression is essential for normal growth and development. Polycomb repressive complexes 1 and 2 (PRC1&2) are involved in this process by establishing monoubiquitination of histone 2A (H2Aub1) and subsequent trimethylation of lysine 27 of histone 3 (H3K27me3). Previous work proposed that H2Aub1 removal by the ubiquitin-specific proteases 12 and 13 (UBP12 and UBP13) is part of the repressive PRC1&2 system, but its functional role remains elusive.

**Results:**

We show that UBP12 and UBP13 work together with PRC1, PRC2, and EMF1 to repress genes involved in stimulus response. We find that PRC1-mediated H2Aub1 is associated with gene responsiveness, and its repressive function requires PRC2 recruitment. We further show that the requirement of PRC1 for PRC2 recruitment depends on the initial expression status of genes. Lastly, we demonstrate that removal of H2Aub1 by UBP12/13 prevents loss of H3K27me3, consistent with our finding that the H3K27me3 demethylase REF6 is positively associated with H2Aub1.

**Conclusions:**

Our data allow us to propose a model in which deposition of H2Aub1 permits genes to switch between repression and activation by H3K27me3 deposition and removal. Removal of H2Aub1 by UBP12/13 is required to achieve stable PRC2-mediated repression.

## Background

Multicellular organisms start as single-celled zygotes progressively develop into organisms with many different cell types. This process requires the function of Polycomb repressive complexes 1 and 2 (PRC1&2) that prevent the differentiation of undifferentiated cells, while in differentiated cells, PRC1&2 prevent dedifferentiation or activation of genes associated with other cell lineages [1, 2]. PRC1&2 repress genes by modifying histone tails: PRC1 adds one ubiquitin moiety to a lysine residue of canonical histone 2A (H2Aub1, on K121 in *Arabidopsis*, analogous to K118 in *Drosophila* and K119 in mammals), and PRC2 adds three methyl groups to lysine 27 of histone 3 (H3K27me3). PRC1&2 ultimately cause transcriptional repression via chromatin compaction [3–5], and other mechanisms remain to be fully explored [2]. At a subset of PRC2 target loci, H2Aub1 needs to be established before H3K27me3 can be deposited, indicating an order of hierarchy with PRC1 acting first and PRC2 after [6, 7]. However, other loci do not require PRC1 prior to being targeted by PRC2 [6, 7]. What determines whether PCR1 is required for PRC2 recruitment remains unclear thus far. Furthermore, there are genes without H2Aub1 despite their targeting by PRC2 depends on PRC1, suggesting that H2Aub1 was initially established and then removed [6].

In plants, removal of H2Aub1 is catalyzed by the ubiquitin-specific proteases 12 and 13 (UBP12 and UBP13) [8]. These proteins interact with LIKE HETEROCHROMATIN PROTEIN 1 (LHP1), which is part of the plant-specific EMBRYONIC FLOWER1 complex (EMF1c) that in addition to EMF1 contains one of the three H3K27me3 readers: LHP1, EARLY BOLTING IN SHORT DAYS (EBS), and SHORT LIFE (SHL) [9]. EMF1, LHP1, EBS, and SHL interact with the PRC1 component BMI1 [9–11], though mutation of *EMF1* and *LHP1* does not result in reduced H2Aub1 levels [6, 12]. EMF1 and LHP1 also interact with the PRC2 subunit MULTICOPY SURPRESSOR OF IRA 1 (MSI1) allowing the maintenance and spreading of H3K27me3 [13–15]. It was hypothesized that the EMF1c has assumed the H3K27me3 spreading and chromatin compaction function of PRC1 in plants [9]. UBP12 and UBP13 have also been shown to be important for the maintenance of H3K27me3 subsequent gene repression, though only at a subset of PRC2 target loci [8]. Similarly, in *Drosophila*, H2A deubiquitinases are genetically connected with Polycomb-mediated repression [16], but the mechanism behind remains unclear. It has been proposed that H2Aub1 is removed from areas of the genome where the modification is not required for repression, making it available for gene repression in other parts of the genome [16]. Alternatively, H2Aub1 could act as a temporary mark that needs to be deposited and subsequently removed from the same loci for stable repression [16]. The present study aimed at testing these hypotheses and to unravel the mechanism by which H2A deubiquitination causes gene repression.

## Results

### UBP12 and UBP13 redundantly repress response genes

UBP12 was previously shown to remove H2Aub1 in vitro and in vivo [8]. The close homolog of UBP12, UBP13 (91% sequence similarity [17]), likely acts redundantly with UBP12, since *ubp12* and *ubp13* single mutants have a wild-type (wt)-like phenotype [18], while a complete knock-out of both genes is not viable [18]. To study the functional requirement of UBP12 and UBP13, we analysed a double mutant with the weak *ubp13-3* allele. This mutant is sterile and severely stunted in growth [18]. In order to determine whether there are UBP12- or UBP13-specific functions, we performed a transcriptome analysis on 2-week-old seedlings of both single mutants and this double mutant. In the single mutants, only a few genes were deregulated, contrasting with a large set of deregulated genes detected in the double mutant (40, 33, and 1128 for *ubp12*, *ubp13*, and *ubp12/13*, respectively, see Additional file 2: Table S1). Nevertheless, in both the single and double mutants, significantly more genes were upregulated than downregulated (Pearson’s chi-square test; *ubp12*: *p* = 9.7E−3; *ubp13*: *p* = 9.2E−9; *ubp12/13*: *p* = 1.7E−102). A Gene Ontology (GO) analysis revealed that most upregulated genes in the double mutant were involved in the response to stimuli (45 out of 60 enriched GO terms, see Additional file 3: Table S2). Most GO terms found in *ubp12* were also identified in the double mutant (13 out of 16 enriched terms), while in *ubp13*, only one GO term was significantly enriched but not found in either *ubp12* or the double mutant. A principal component analysis (PCA) on the transcriptome data showed that the single mutants clustered together with the wt and away from the double mutant, supporting the idea of genetic redundancy (Additional file 1: Figure S1). Together, our data show that UBP12 and 13 have a largely redundant function in the repression of response genes and are henceforth collectively called UBP12/13.

### UBP12/13 work together with PRC1, PRC2, and EMF1c

**Fig. 1:**
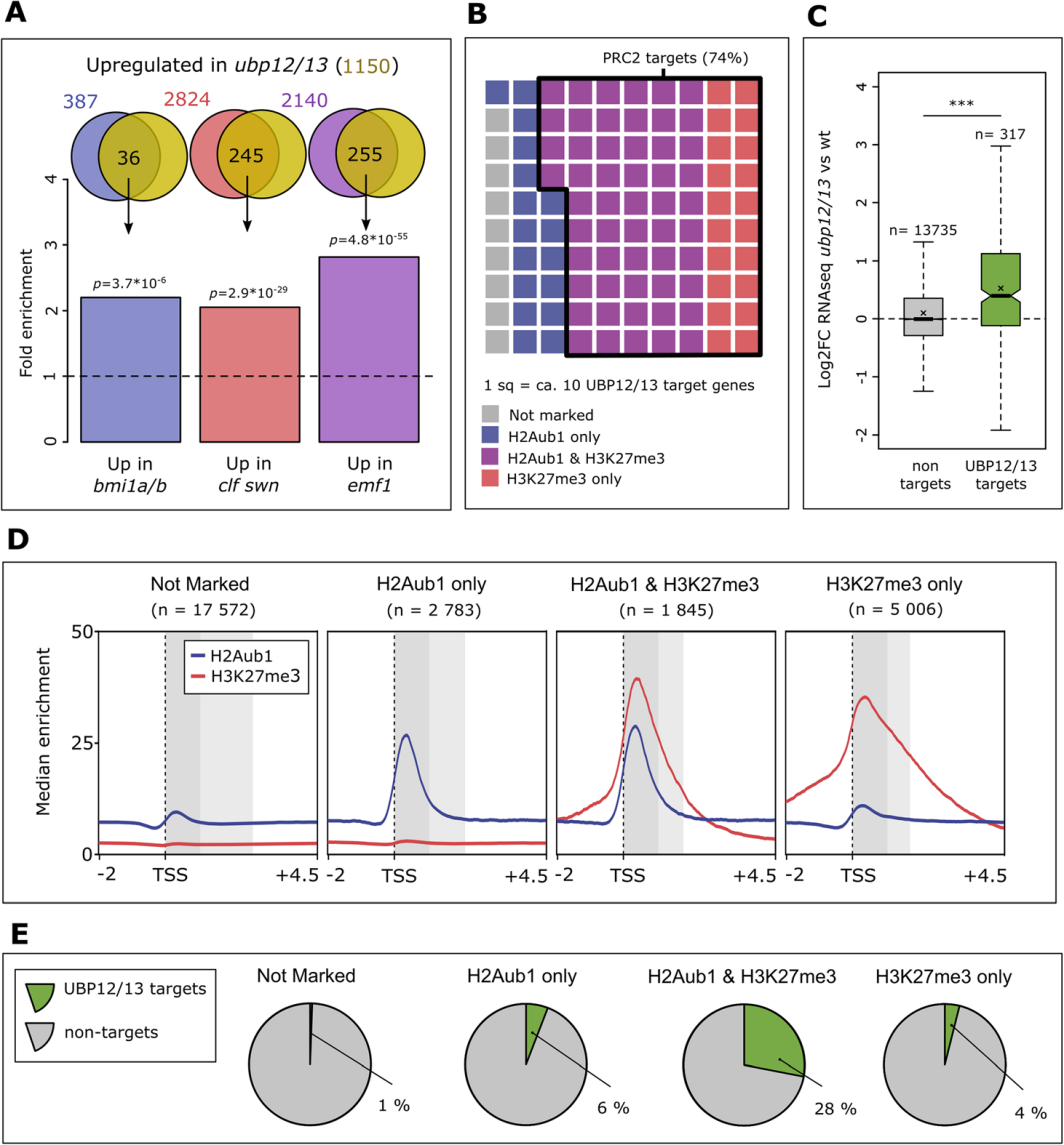
UBP12/13 directly repress PRC2 target genes. **a** Fold enrichment of the overlap of lists of genes upregulated in *ubp12/13* (1150 genes, yellow circle) and genes upregulated in PRC1 (*bmi1a/b*, 387 genes, blue circle) [19], PRC2 (*clf swn*, 2824 genes, red circle) [19], and EMF1c (*emf1*, 2140 genes, purple circle) [9] mutants. The dashed line indicates the expected overlap based on chance. *p* values are based on a hypergeometric test for significant over-enrichment. **b** Waffle plot showing the presence of the H2Aub1 and H3K27me3 marks in wild type on UBP12/13 target genes. The black border indicates those UBP12/13 targets that are marked with H3K27me3 (and hence PRC2 targets). Each square corresponds to about 10 genes. **c** Boxplots showing differential expression of all expressed UBP12/13 targets (green) and all other protein-coding genes (grey). Test for significance was done by the Mann-Whitney *U* test; ns *p* ≥ 0.05, *p < 0.05, **p < 0.01,***p < 0.001. **d** Metagene profiles showing the median of H2Aub1 and H3K27me3 enrichment for gene categories defined by the presence of significant H2Aub1 and H3K27me3 peaks. The darker grey box indicates the first 1 kb of the gene body, and both grey boxes together indicate the mean gene body size within that category. **e** Pie charts indicating the percentage of genes in the indicated category corresponding to UBP12/13 targets. Note that above “UBP12/13 targets” refers to genes gaining H2Aub1 in *ubp12/13* mutants. The biological material underlying the RNA-seq data in this figure is 2-week-old whole plants (*bmi1a/b*, *clf swn*, and *ubp12/13*) and 3-week-old whole plants (*emf1*). ChIP-seq data was obtained from 33 DAG rosettes

Previous research indicated that UBP12/13 work together with PRC2 to maintain H3K27me3 and repress gene expression [8]. However, the molecular mechanism by which UBP12/13 achieve this function remained unclear. To address this question, we tested whether genes being upregulated in mutants for PRC1, PRC2, or EMF1c complexes were also upregulated in *ubp12/13* mutants. We found that indeed genes upregulated in PRC1, PRC2, and EMF1c mutants were more likely to be upregulated in *ubp12/13* than expected by chance (Fig. 1a), supporting the hypothesis that UBP12/13 are working together with PRC1/PRC2/EMF1c to repress genes.

### UBP12/13 repress PRC2 targets via H2Aub1 deubiquitination

To elucidate the mechanism by which UBP12/13 cause gene repression, we identified genes targeted by UBP12/13 by performing H2Aub1 chromatin immunoprecipitation followed by sequencing (ChIP-seq) on 33 DAG rosette leaves of the *ubp12/13* mutant and wt. Surprisingly, we identified a larger number of genes that lost H2Aub1 in the *ubp12/13* mutant than those that gained it (11% vs 3%, respectively). Since UBP12/13 are deubiquitinases, this was rather unexpected. The global loss of H2Aub1 was confirmed by western blotting (Additional file 1: Figure S2). We focussed further analyses on loci gaining H2Aub1, as this is expected to be a direct consequence of lack of UBP12/13. The majority of regions gaining H2Aub1 were genes, only 13% of peaks fell outside of genic regions, comprised by the gene bodies plus 2 kb upstream of the TSS (Additional file 4: Table S3). Because H2Aub1 and H3K27me3 are usually the highest enriched in the first 1 kb of the gene body [6], we focussed subsequent analyses on this region. We found that 68% of increased H2Aub1 peaks were located in this region and that 3% of protein-coding genes had increased H2Aub1 in this area (for comparison, 17% of protein-coding genes were marked with H2Aub1 in wt). Because UBP12/13 are deubiquitinases, we considered the 3% of genes gaining H2Aub1 in the *ubp12/13* mutant as direct UBP12/13 targets (see Additional file 5: Table S4) and will be referred to as such from here on.

To elucidate the connection between UBP12/13 and PRC2 function, we determined which genes were marked by H3K27me3 and H2Aub1. We categorized genes based on the presence of these marks in the first 1 kb of the gene body as ‘not marked’ (lacking either mark), ‘H2Aub1 only’, ‘H2Aub1 and H3K27me3’, and ‘H3K27me3 only’ (see Additional file 6: Table S5 for gene lists and Fig. 1d for metagene profiles). Out of 960 UBP12/13 target genes, 74% were marked with H3K27me3 (irrespective of H2Aub1 presence, Fig. 1b). We thus conclude that most loci subject to UBP12/13-mediated H2A deubiquitination are PRC2 targets.

The majority (77%) of deregulated genes in *ubp12/13* double mutants was upregulated (Additional file 2: Table S1), strongly suggesting that UBP12/13 function in gene repression. This view is supported by the fact that on average, direct UBP12/13 target genes were upregulated, while non-targets, were not (Fig. 1c, Additional file 1: Figure S3). We thus conclude that UBP12/13 have a direct role in the repression of PRC2 target genes.

### The repressive role of H2Aub1 depends on PRC2

**Fig. 2:**
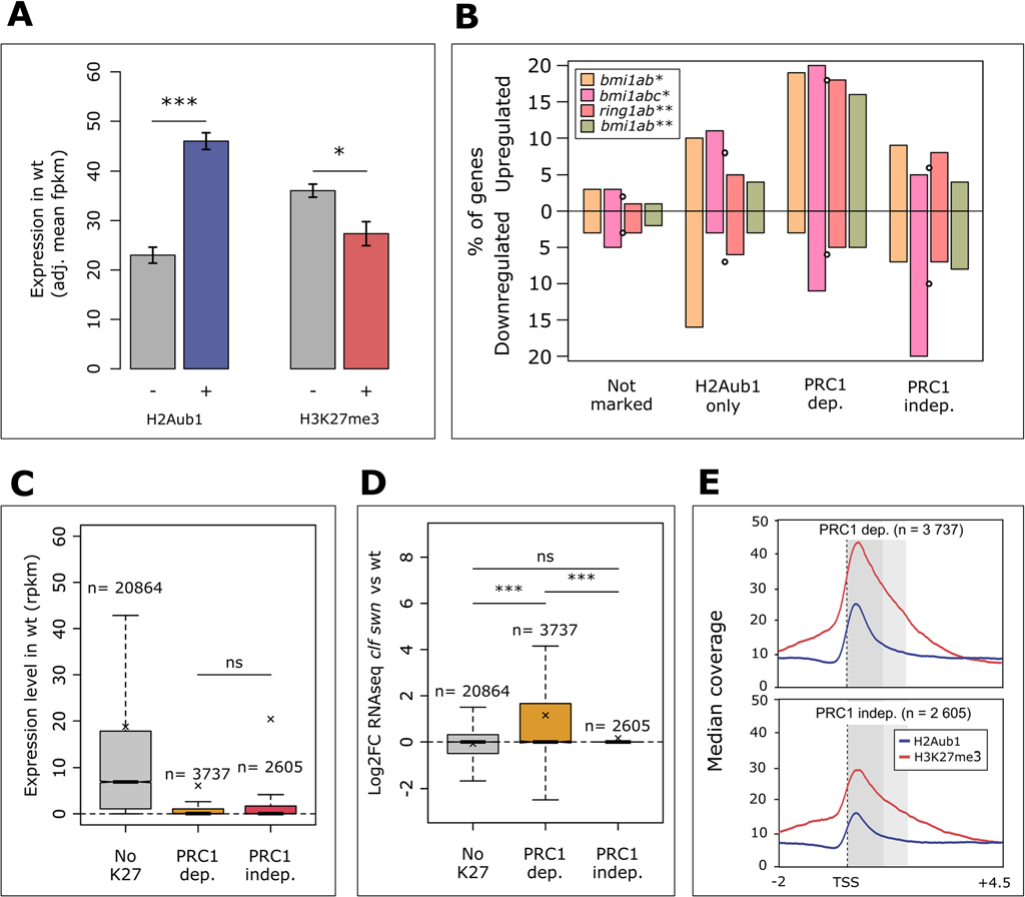
H2Aub1 is not a repressive mark per se. **a** Mean wild-type (wt) expression in the absence (−) or presence (+) of H2Aub1 or H3K27me3 after correction for the presence of the other mark (ANCOVA estimated marginal means, in fpkm). **b** Percentage of upregulated and downregulated genes in PRC1 mutants among the four categories defined by the presence of H2Aub1 and H3K27me3 and dependency of H3K27me3 on PRC1. Asterisks indicate the |log2FC| cut-off used to call significant deregulation: *≥ 4 or **≥ 1; the *p* value cut-off was 0.05 for both. Small circles indicate the mean of the four mutants. **c** wt expression level for genes defined by the presence of H3K27me3 and PRC1 dependency. **d** Expression change in a PRC2 (*clf swn*) mutant compared to wt for genes defined by the presence of H3K27me3 and PRC1 dependency. **e** Metagene profiles of H3K27me3 and H2Aub1 in PRC1-dependent and PRC1-independent gene categories. Based on re-analysed ChIP-seq data from [6] (**a**–**e**) and RNA-seq data from [20] (**a**, b*) and [19] (b**, **c**, **d**). An ANCOVA post hoc test was used to test for significance in **a**, and a Mann-Whitney *U* test for **c** and **d**; ns *p* ≥ 0.05, *p < 0.05, **p < 0.01,***p < 0.001; Bonferroni correction was applied with *m* = 6. The biological material underlying this figure is whole plants of 7 DAG (ChIP-seq data), 10 DAG (RNA-seq data in **a** and b*), and 14 DAG (RNA-seq data in b**, **c**, **d**)

Our data revealing that H2Aub1 removal by UBP12/13 is necessary for repression contrasts with the idea that H2Aub1 applied by PRC1 is a repressive mark per se. Supporting this notion, previous research revealed that genes with only H3K27me3 are more likely to be repressed than genes marked with both H2Aub1 and H3K27me3 [6]. To gain further insight into the role of H2Aub1, we determined the mean expression level of genes with or without H2Aub1 and H3K27me3 in the first 1 kb of the gene body using an analysis of covariance (ANCOVA) (Fig. 2a). This analysis revealed that H2Aub1 has a positive association with expression, while confirming the expected negative association between H3K27me3 and expression. To further unravel the relationship between PRC1-mediated H2Aub1 deposition and gene repression, we compared the differential expression in several PRC1 mutants between four non-overlapping gene categories defined by the presence of H2Aub1 and H3K27me3 (re-analysed data from [19, 20]). Genes with H3K27me3 were subdivided based on their PRC1 dependency for H3K27me3 establishment (instead of H2Aub1 presence). Genes that had decreased H3K27me3 within the first 1 kb in the PRC1 mutant *bmi1a/b/c* were considered PRC1-dependent (PRC1-dep), and all other H3K27me3-marked genes were considered PRC1-independent (PRC1-indep) (Additional file 7: Table S6). Data from *bmi1a/b*, *bmi1a/b/c*, and *ring1a/b* mutants was used in the analysis. The core of PRC1 in animals is a heterodimer of a RING1 class protein and a BMI1 class protein, with RING1 possessing the E3 ubiquitin ligase activity and BMI1 being an important assisting factor [21, 22]. *Arabidopsis* RINGs and BMI1s both have E3 activity in vitro, and previous research showed that *bmi1a/b/c*, and even *bmi1a/b*, lacks most H2A ubiquitination [6, 10]. The *ring1a/b* double mutant is also expected to lack H2A ubiquitination, but this remains to be tested. For these different mutants, we examined percentages of significantly up- and downregulated genes for the aforementioned categories and found that only PRC1-dependent genes had a consistently higher percentage of upregulated genes than downregulated genes in PRC1 mutants, and on average the highest percentage of upregulated genes (Fig. 2b). This reveals that PRC1-mediated repression depends on H3K27me3 and that H2Aub1 is not necessarily associated with repression. Further analysis of all genes in a *bmi1a*/*b* double mutant showed a similar pattern (Additional file 1: Figure S4). Together, our data reveal that H2Aub1 unlikely has a direct repressive effect, rather it can repress genes indirectly by recruiting PRC2.

We next addressed the question why certain genes require PRC1 and H2Aub1, while other genes can be targeted by PRC2 independently of PRC1. PRC1-dependent and independent genes marked by H3K27me3 did not differ in their expression level in wt (Fig. 2c); however, PRC1-dependent genes were significantly stronger upregulated in a PRC2 mutant background compared to PRC1-independent genes (Fig. 2d, Additional file 1: Figure S5, also see Fig. 2e for metagene profiles for PRC1-independent and dependent genes). This suggests that PRC1-independent H3K27me3-marked genes are already repressed before PRC2 recruitment (and so before acquiring H3K27me3), differing from PRC1-dependent genes that require PRC2 (and H3K27me3) for repression. To test the connection between PRC1 and UBP12/13, we overlapped UBP12/13 targets with PRC1-independent, PRC1-dependent, and non-K27-marked gene lists. We found that PRC1-dependent genes are preferentially targeted over PRC1-independent genes (Additional file 1: Figure S6). Together, we conclude that PRC1 likely recruits PRC2 to active genes to mediate their repression, while PRC1 appears dispensable for the PRC2 recruitment to inactive genes.

### Gain of H2Aub1 in *ubp12/13* is associated with loss of H3K27me3

**Fig. 3:**
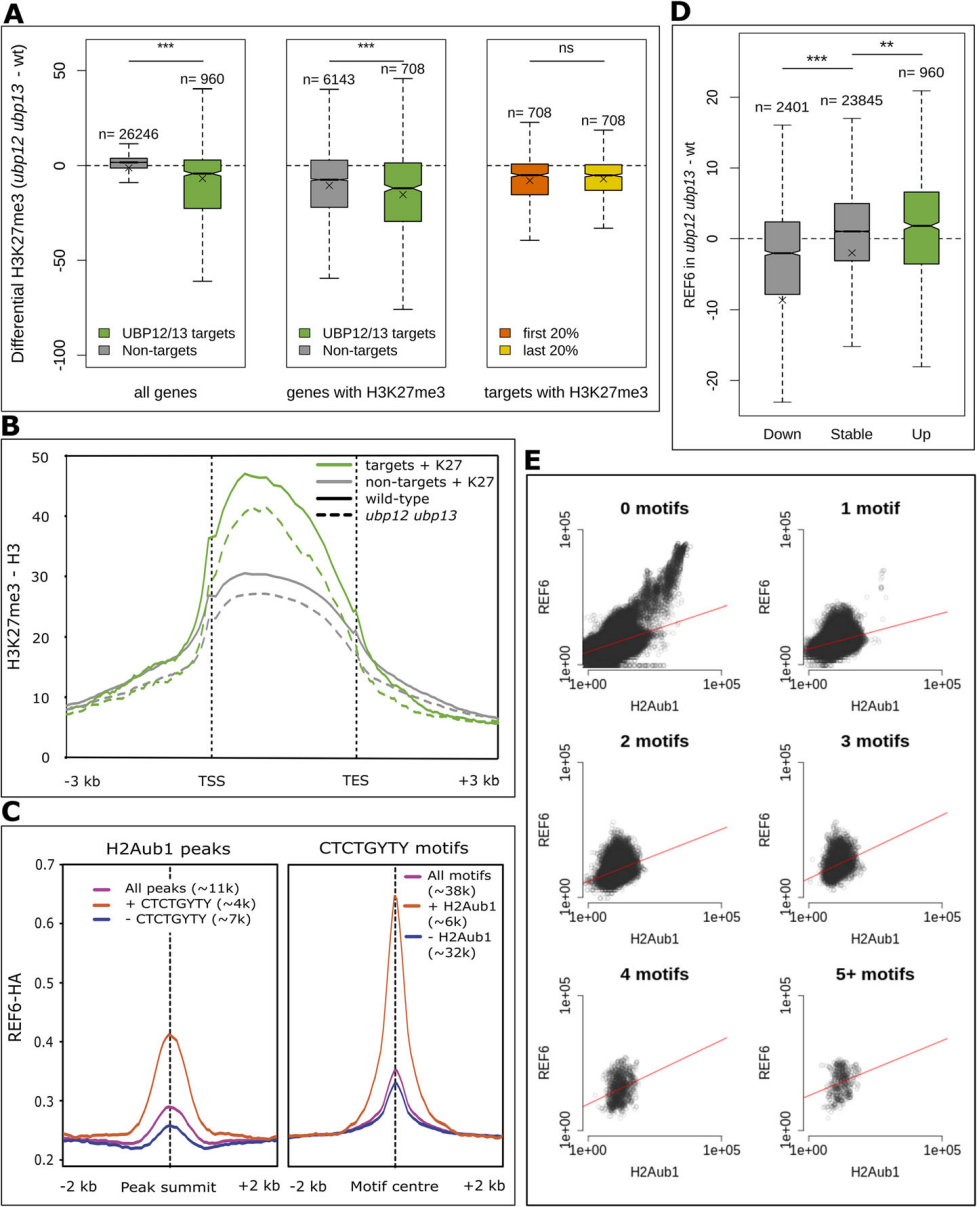
UBP12/13 protect against H3K27me3 removal. **a** Differential H3K27me3 enrichment in the *ubp12/13* mutant minus wild type, for UBP12/13 targets and non-targets (left), for UBP12/13 targets and non-targets marked with H3K27me3 (middle), and for UBP12/13 targets with H3K27me3 on the first 20% or the last 20% of gene bodies (right). Tests for significance (for **a**–**c**) were done by Mann-Whitney *U* test; ns *p* ≥ 0.05, *p < 0.05, **p < 0.01,***p < 0.001; Bonferroni correction was applied with *m* = 6. **b** Metagene plot showing the median H3K27me3 coverage-scaled fragment counts in the *ubp12/13* mutant (dashed line) and wild type (solid line), for UBP12/13 targets with H3K27me3 (green) and non-targets with H3K27me3 (grey). **c** REF6 enrichment (median coverage-scaled fragment count) at narrow H2Aub1 peaks (left) and at CTCTGYTY motifs (right). Depicted in the left panel are all significant H2Aub1 peaks (purple), all peaks with at least one CTCTGYTY motif (red) and all peaks that do not possess at least one CTCTGYTY motif (blue). Depicted in the right panel are all CTCTGYTY motifs (purple), all motifs with H2Aub1 (red), and all motifs without H2Aub1 (blue). REF6-HA binding sites and CTCTGYTY data obtained from [25]. **d** Differential coverage-scaled REF6 fragment counts (mutant-wt) for genes that lose H2Aub1 (“Down”), genes without a change in H2Aub1 (“Stable”), and genes gaining H2Aub1 (“Up”). **e** Scatterplots made with the coverage-scaled fragment counts of H2Aub1 and REF6 in 50-bp windows (genome-wide). CTCTGYTY motifs were also counted in 600-bp windows around the centre of the windows, and windows were split over separate plots depending on the number of motifs. The *x*- and *y*-axes are logarithmic (Log10). Statistics: 0 motifs (*m* = 0.33, *R*^2^ = 0.17, *p* < 2.2E−16), 1 motif (*m* = 0.27, R^2^ = 0.09, *p* < 2.2E−16), 2 motifs (*m* = 0.39, *R*^2^ = 0.11, *p* < 2.2E−16), 3 motifs (*m* = 0.47, *R*^2^ = 0.10, *p* < 2.2E−16), 4 motifs (*m* = 0.47, *R*^2^ = 0.07, *p* < 2.2E−16), and 5+ motifs (*m* = 0.41, *R*^2^ = 0.05, *p* < 3.7E−9). The biological material used to generate the data in this figure was 33 DAG rosettes, apart from the REF6-HA data, which was obtained from 12 DAG seedlings

We had previously shown that some PRC2 targets lose H3K27me3 in a *ubp12/13* mutant [8]. To test the connection between H3K27me3 and UBP12/13 function on a genome-wide level, we analysed H3K27me3 enrichment in *ubp12/13* compared to wt for UBP12/13 direct targets as well as non-targets. Direct UBP12/13 targets had significantly decreased H3K27me3 levels compared to non-targets (Fig. 3a, left). When only considering UBP12/13 targets and non-targets marked with H3K27me3, we similarly found that UBP12/13 targets displayed a significantly stronger loss of H3K37me3 than non-targets (Fig. 3a, middle) implicating UBP12/13 in the maintenance of H3K27me3. The global loss of H3K27me3 could be an indirect effect of downregulation of PRC2 or EMF1c components in *ubp12/13* mutants. However, mRNA level of the analysed PRC1 and PRC2 subunits was not changed (*GENE*: Log2FC, *p*_adj_—*SWN*: 1.5E−1, 8.2E−1; *CLF*: − 4.5E−1, 6.9E−1; *MEA*: ND; *FIE*: ND; *MSI1*: 2.0E−1, 8.8E−1; *FIS2*: ND; *EMF2*: − 8.7E−2, 9.4E−1; *VRN2*: 3.7E−1, 5.2E−1; *EMF1*: 7.2E−2, 9.5E−1; *EBS*: 2.1E−2, 9.9E−1; *SHL*: − 1.8E−3, 1.0; *LHP1*: 2.9E−2, 9.9E−1). The loss is also unlikely an artefact of faulty ChIP-seq normalization, as the same general loss was observed by western blot (Additional file 1: Figure S7). In the *lhp1* mutant, H3K27me3 is lost only at the 3′ end of the gene body, indicating that LHP1 is involved in H3K27me3 spreading [14, 15]. Because UBP12/13 interact with LHP1 [8], we tested whether loss of H3K27me3 in *ubp12/13* occurs predominantly at the 3′ end of the genes. We found that loss of H3K27me3 in *ubp12/13* occurs similarly on both ends of the gene (Fig. 3a, right panel). This, together with the fact that H3K27me3 enrichment at UBP12/13 targets is higher than at non-targets in wt (Fig. 3b), indicates that UBP12/13 do not promote H3K27me3 spreading, but rather prevent loss of H3K27me3. Loss of H3K27me3 in *ubp12/13* could be a consequence of reduced deposition or increased removal of H3K27me3. Since recruitment of PRC2 on about two thirds of genes depends on PRC1, we considered it rather unlikely that the presence of H2Aub1 negatively interferes with PRC2 recruitment. We tested the alternative hypothesis that loss of H3K27me3 in *ubp12/13* is a consequence of H3K27me3 being removed from genes with H2Aub1, while genes without H2Aub1 are protected. Proteins of the KDM4/JMJD2 family function as H3K27me3 demethylases in *Arabidopsis* [23–25]. By re-analysing previously published ChIP-seq data [25], we indeed found that the KDM4 RELATIVE OF EARLY FLOWERING 6 (REF6) binds to and removes H3K27me3 preferentially from genes marked with H2Aub1 (Additional file 1: Figure S8 and S9). We then asked whether H2Aub1 could recruit REF6. It was previously shown that REF6 binds the CTCTGYTY motif; however, since only about 15% of the wide-spread CTCTGYTY motifs are within REF6-bound regions, a single motif is not a good predictor for REF6 recruitment [25]. As reported in a previous study [25], clusters of four or more motifs are frequently (94%) bound by REF6, but the majority (93%) of REF6-positive regions does not have such a motif cluster (Additional file 1: Figure S10), indicating that additional factors assist in the recruitment of REF6. We used previously published REF6 ChIP-seq data [25] and our H2Aub1 data to determine whether REF6 is enriched at H2Aub1 peak summits (Fig. 3c, left panel). We found that REF6 is indeed enriched at these peaks, though the level was rather low for peaks that do not contain at least one motif. We also examined the REF6 level at CTCTGYTY motifs and found that the presence of H2Aub1 increased the median enrichment level by a factor of two compared to motifs without H2Aub1, indicating H2Aub1 is indeed required for REF6 recruitment (Fig. 3c, right panel). To test whether REF6 can be indeed recruited by H2Aub1 and is not merely co-localized for another reason, we performed REF6 ChIP-seq using REF6-GFP lines previously described [24] (Additional file 8: Table S7). Our newly generated data confirmed the results we obtained with the published data (Figure S11 and S12). Next, we analysed the differential REF6 enrichment in the *ubp12/13* mutant compared to wild type, for genes gaining, losing, or having unchanged H2Aub1 in the mutant (Fig. 3d). We found that genes that lose H2Aub1 also lose REF6, and genes gaining H2Aub1 also gain REF6. This confirms the hypothesized role of H2Aub1 in the recruitment of REF6. Lastly, we wondered how H2Aub1 and CTCTGYTY motifs work together to recruit REF6, so we determined the H2Aub1 and REF6 enrichment values in 50-bp windows across the genome, as well as the numbers of CTCTGYTY motifs in 600 bp around the centres of those windows, and made scatterplots with the resulting data (Fig. 3e). We found that in general, H2Aub1 correlated well with REF6 (slope between 0.27 and 0.47), regardless of the number of motifs. Together, we propose that UBP12/13 maintain H3K27me3 via protection against demethylation.

### H2Aub1 is associated with responsiveness

Our data suggest that H2Aub1 is not a repressive mark per se, but that it can cause repression via PRC2-mediated H3K27me3 deposition (Fig. 2), while at the same time, it has an anti-repressive role possibly due to the recruitment of H3K27-demethylases (Fig. 3). Because genes upregulated in *ubp12/13* and genes co-targeted by BRAHMA and REF6 [24] are linked to stimulus response, we hypothesized that H2Aub could provide a standby mode between stable activation and stable repression, allowing genes to quickly respond to stimuli. To test this hypothesis, we performed a GO analysis on genes marked with H2Aub1, as well as genes with H3K27me3, and genes with neither mark. For genes marked with H2Aub1, about half of the significantly enriched GO terms were related to stimulus response (33/76), while none (0/26) or only one (1/67) enriched GO term for stimulus response was found for genes with H3K27me3 or genes without either mark, respectively (see Additional file 9: Table S8). Next, we tested whether genes with H2Aub1 have a higher potential to change their expression than genes without this modification. We re-analysed previously published ‘total gene responsiveness’ scores based on microarray data from 467 conditions [26] (see Additional file 10: Table S9). Total gene responsiveness is defined by the number of conditions in which a gene is deregulated compared to the control condition. Based on this definition, housekeeping genes and constitutively repressed genes have a low score, while hypervariable genes have a high score [24]. We found that the presence of H2Aub1 is positively linked to responsiveness and that the H3K27me3 mark shows a weak negative association (Additional file 1: Figure S13). Taken together, our results support the idea that H2Aub1 provides a stand-by state for rapid expression change.

### Role of H2A.Z in H3K27me3 maintenance

It was recently shown that H2A.Z can be monoubiquitinated [27]. Since the commonly used H2Aub1 antibody, which was used in this study, also reacts with monoubiquitinated H2A.Z [27], we tested whether H2A.Zub1 is targeted by UBP12/13. We performed H2A.Z ChIP-seq (Additional file 11: Table S10) and analysed the overlap of UBP12/13 targets and H2A.Z-marked genes. We found that most (93%) of UBP12/13 targets contain H2A.Z, which was significantly higher (1.2×) than based on random chance (Additional file 1: Figure S14A). Using previously published data [27], we asked whether UBP12/13 targets were enriched for putative H2A.Zub1-marked genes. We identified deregulated genes in a mutant line expressing an H2A.Z variant that cannot be ubiquitinated (*hta9* HTA-RR). We found that UBP12/13 targets were strongly enriched for upregulated genes in the substitution line (Additional file 1: Figure S14B), consistent with the proposed role of H2A.Zub1 in gene repression [27]. We next tested whether H2A.Z has any effect on differential gene expression in the *ubp12/13* mutants. However, we found that UBP12/13 targets with and without H2A.Z were similarly upregulated (Additional file 1: Figure S15), suggesting that the repressive effect mediated by UBP12/13 is not specific for H2A.Z containing nucleosomes. Previous work established that there is no global loss of H3K27me3 in mutants depleted for H2A.Z [27], contrasting with the loss of H3K27me3 observed on UBP12/13 targets in *ubp12/13* mutants. We thus conclude that the repressive effect mediated by UBP12/13 is unlikely to be connected to H2A.Z, consistent with the proposed independency of H2A.Z-mediated repression and PRC2 function [27].

## Discussion

Our study revealed that UBP12 and 13 work together with PRC1, PRC2, and EMF1 to repress genes involved in stimulus response. We found that UBP12/13 deubiquitinate H2A at PRC2 target genes and that this is associated with repression of those genes. We also observed that these genes lose H3K27me3 in the *ubp12/13* mutant, revealing that UBP12/13 activity is required to maintain correct H3K27me3 levels. The mechanism of how the removal of H2Aub1 could lead to gene repression has been enigmatic, because removal of PRC1 causes PcG target gene activation [8, 16]. Our data provide an answer to this question, by revealing that PRC1/H2Aub1 does not cause repression by itself but allows recruitment of PRC2 and that H2Aub1 aids in the recruitment of the H3K27me3 demethylase REF6. Thus, removal of H2Aub1 prevents gene reactivation by preventing active H3K27me3 demethylation.

We have shown that UBP12/13 targets are involved in response processes, as are H2Aub1-marked genes in general. Responsive genes are also targeted by REF6 [24], consistent with the finding that REF6 is enriched at genes marked with H2Aub1. Similarly, it has been proposed that active H3K27me3 demethylation by KDM6 family members JMJD3 and UTX in mammals is required for stimulus response [28]. Mammalian JMJD3 has been shown to be upregulated in different cell types in response to inflammatory signals and hypoxia [29–32], and in *Caenorhabditis elegans*, JMJD3 is required for the heat shock response [33]. Furthermore, in mammalian macrophages, JMJD3 is preferentially targeted to bivalent genes [29], i.e. genes marked with both H3K4me3 and H3K27me3, which are inactive but can be rapidly activated [34]. And in mammalian stem cells, UTX is required for the activation of retinoic acid-inducible bivalent genes [35]. Together with our data, this strongly suggests that H3K27me3 demethylation in responsive genes is conserved across kingdoms.

Our data furthermore revealed that there are two distinct modes of PRC2 recruitment: a PRC1-dependent recruitment to expressed genes and a PRC1-independent recruitment to silent genes. Also in *Drosophila*, distinct sets of genes differ in their requirement of PRC1 for PRC2 recruitment and about a third of PcG targets recruit PRC2 independently of PRC1 [36]. What determines whether a gene requires PRC1 for PRC2 recruitment has not yet been revealed. However, consistent with our model, it has been speculated that PRC1-independent targets cannot be switched to a transcriptionally active state, while PRC1-dependent genes are susceptible to the action of Trithorax-group proteins and thus potentially active [7]. Also consistent with this model is the fact that animal PRC1 binds at many transcriptionally active genes [37–39], possibly enabling switching to the transcriptionally repressed state. Finally, it has been shown that PRC2 binds nascent RNA non-specifically, and it has been proposed that RNA binding prevents PRC2 from depositing H3K27me3 [40–42]. Therefore, the requirement of PRC1 to enable PRC2 recruitment to active genes could be conserved between animals and plants.

In addition to H2A deubiquitination, UBP12/13 were reported to remove polyubiquitin from the transcription factor MYC2 in vitro, causing its stabilization [43]. Furthermore, the receptor-like kinase RGFR1 was stabilized when overexpressing UBP13 [44], suggesting that UBP12/13 have additional functions. Nevertheless, our study concentrated on genes gaining H2Aub1 in *ubp12/13* mutants, which is likely a consequence of UBP12/13 acting as histone deubiquitinase. In addition to the genes that gained H2Aub1, we unexpectedly observed twice as many that lost H2Aub1. One possible explanation is that the lack of removal of H2Aub1 from UBP12/13 targets limits the amount of available ubiquitin on other targets. Another explanation is that PRC1 function is compromised. While no gene encoding for PRC1 subunits was downregulated in *ubp12/13*, it remains possible that UBP12/13 are required to stabilize PRC1 subunits at the protein level. The mammalian homolog of UBP12/13, USP7, deubiquitinates and thereby stabilizes a subunit of PRC1 [45–47], and a similar scenario may apply to plants. This could potentially cause an underestimation of the number of loci on which H2Aub1 is removed, which, however, should not affect the general conclusions drawn from this work.

Whether a similar mode of repressive H2A deubiquitination mediated by UBP12/13 exists in animals is unclear. Animal USP7 does not target H2A, but rather H2B [48–50]. In *Drosophila*, Calypso is an unrelated protein that was proposed to have a similar role to UBP12/13, as it is required for the repression of PRC2-targeted *HOX* genes [51]. However, the mammalian homolog of Calypso, BAP1, has recently been shown to remove H2Aub1 in order to activate genes [52]. BAP1 targets do not possess H3K27me3 in wt, but do in the *bap1* mutant, indicating that BAP1 prevents recruitment of PRC2 by PRC1 [52]. This function of BAP1 does however not preclude the existence of repressive H2A deubiquitination in animals, as other H2A deubiquitinases could have that role [53].

## Conclusions

**Fig. 4:**
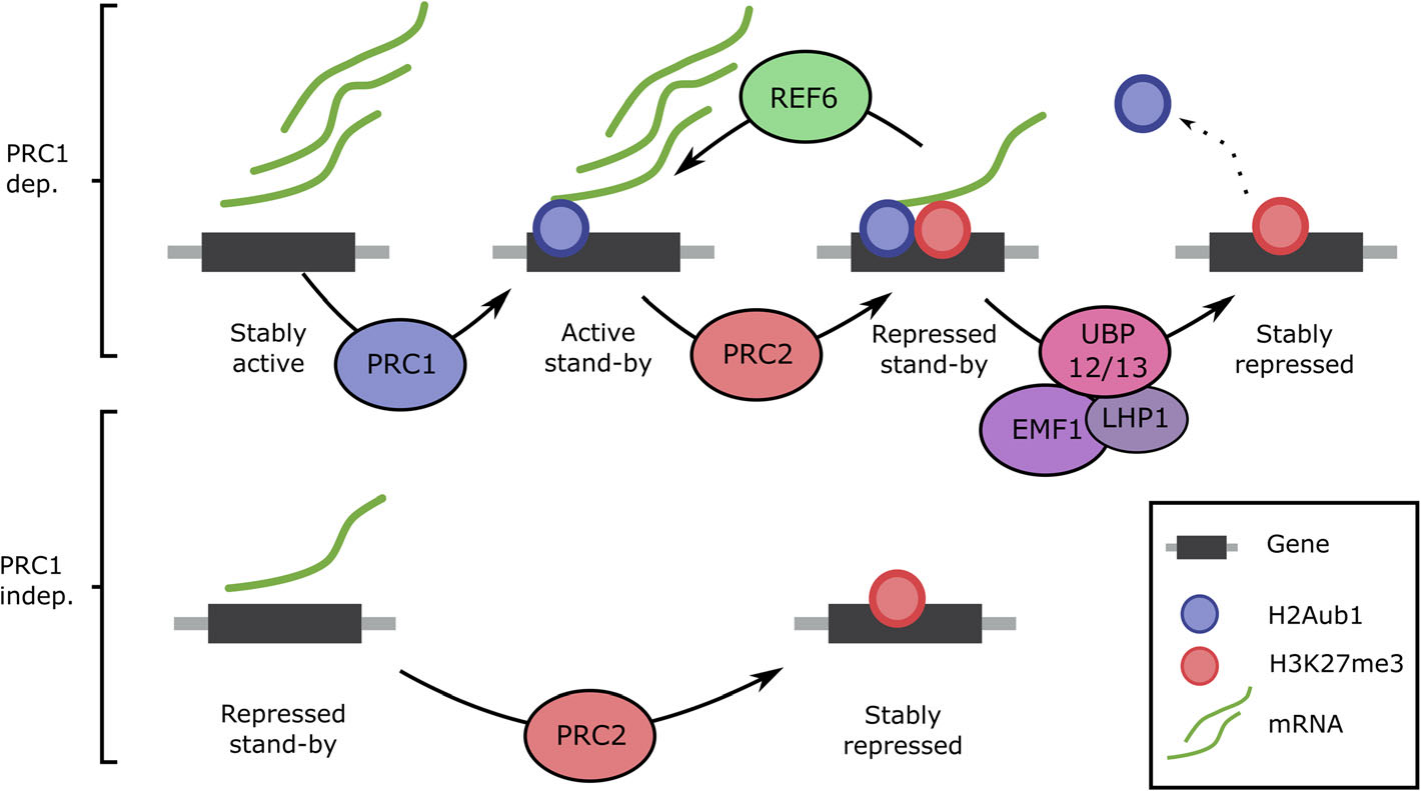
Model of PRC1/PRC2-mediated gene regulation. Before PRC1/PRC2 recruitment, PRC1-dependent genes are active, while PRC1-independent genes are already pre-repressed. The pre-repressed genes can be directly targeted by PRC2, while PRC2 targeting to active genes is inhibited, possibly by nascent RNA. PRC1 presence mitigates this inhibition, allowing PRC2 to target and silence the genes. However, PRC1-mediated H2Aub1 enhances REF6 binding and H3K27 demethylation, allowing genes marked by H2Aub1, to be rapidly reactivated in response to a stimulus. Therefore, removal of H2Aub1 by UBP12/13 is necessary for stable repression

In summary, based on the data generated in this study, we propose the following model explaining the requirement of PRC1 and UBP12/13 for PRC2-mediated gene repression (Fig. 4): PRC1-independent PRC2 targets have a minimal expression level and can be directly stably repressed by PRC2. In contrast, PRC1-dependent genes are actively expressed; their nascent RNA plausibly inhibits PRC2 function ([40–42]). Alternatively, PRC2 recruitment could be inhibited by transcription factors occupying PRC2-recruiting DNA elements [54]. PRC1 mitigates this inhibition not by causing repression, but more likely by counter-balancing the inhibition by facilitating PRC2 recruitment, possibly by recruiting LHP1 or EMF1 ([10, 11]). In this way, PRC1/H2Aub1 allows PRC2-mediated gene repression, but at the same time, H2Aub1 allows the recruitment of an H3K27me3 demethylase. This allows H2Aub1-marked genes to be rapidly switched between active and repressed states in response to defined stimuli. Stable repression, therefore, requires that after PRC2 recruitment H2Aub1 is removed again by UBP12/13.

## Methods

### Plant material

Mutant alleles used in this study are *ubp12-1* (GABI_244E11) [17, 18], *ubp13-3* (SALK132368) [6], and *ubp12-2w* (GABI_742C10) [55]. Mutant and wt (Col-0) plants were grown under long-day conditions (16 h light, 8 h dark), at 22 ± 2 °C. Because the *ubp12-1 ubp13-3* double mutant is sterile, it was maintained heterozygous for *ubp13-3*. REF6-GFP *ref6* seeds were kindly provided by [24], and plants were crossed to *ubp12-1 ubp13-3* to obtain REF6-GFP *ref6 ubp12-1 ubp13-3* plants. *ubp12-1 ubp13-3* mutants were named *ubp12/13* throughout most of the text. Homozygotes were identified by genotyping and confirmed by phenotyping, see Additional file 12: Table S11 for primers used in this study.

### Antibodies

Antibodies used were anti-H3 (07-690, Merck Millipore, Burlington, MA, USA), anti-H2Aub1 (#8240S, Cell Signaling Technology, Danvers, MA), anti-HTA9 (AS10 718, Agrisera, Vännäs, Sweden), anti-H3K27me3 (07-449, Merck Millipore), anti-H4 (05-858, Merck Millipore), and anti-GFP (A11122, Thermo Fisher Scientific, Waltham, MA, USA).

### Histone extraction and western blotting

Histone extraction and western blotting were performed as previously described [8], with the following modifications: for H2Aub1 western blot, 2-week-old whole *ubp12-2w ubp13-3* seedlings were used, and for H3K27me3 western blot, ca. 30-day-old *ubp12-1 ubp13-3* rosettes were used.

### RNA sequencing

For RNA sequencing, *ubp12-1*, *ubp13-3*, *ubp12-1 ubp13-3*, and wt plants were grown for 2 weeks on half-strength Murashige and Skoog medium (1/2 MS) medium, and whole seedlings were harvested between 6 and 8 h after dark to light transition. RNA was isolated with the MagJET plant RNA purification kit (Thermo Fisher Scientific), and libraries prepared with the TRUseq RNA sample preparation kit v2 (Illumina, San Diego, CA, USA). 50-bp single-end read sequencing was performed on the Hiseq2000 platform (Illumina) at SciLifeLab (Uppsala, Sweden). The RNA-seq experiment was done with three biological replicates per genotype.

### H2Aub1, H2A.Z, H3K27me3, and REF6 ChIP-seq

For H2Aub1, H2A.Z, and H3K27me3 ChIP-seq, *ubp12-1 ubp13-3* and wt plants were grown for 2 weeks on ½ MS medium, then transferred to soil where they grew until the age of 33 days, and then rosette leaves were harvested 1 h before darkness. For REF6 ChIP-seq, we used double mutant and wt plants harbouring a REF6-GFP construct. ChIP was performed as described before [56], with the following modifications: no vacuum infiltration with DMA was performed. Vacuum infiltration with formaldehyde was performed for 2 × 5 min for H2Aub1/H2A.Z and H3K27me3 experiments and 2 × 10 min for the REF6 experiment. For quenching the formaldehyde, the solution was poured off, after which 0.125 M glycine was added and another 5-min vacuum infiltration was performed. Vacuum infiltration was performed at room temperature. Around 300, 500, and 200 mg cross-linked plant material was used for the nuclei extraction for the H2Aub1/H2A.Z, H3K27me3, and REF6, respectively. Sonication of the chromatin was done for eight 30-s ON, 30-s OFF cycles. Overnight antibody binding was performed directly after sonication, followed by the addition of washed protein A Dynabeads (Thermo Fisher Scientific) to each ChIP aliquot. For bead washing, the LiCl wash step was skipped. De-crosslinking and subsequent DNA recovery steps performed using the Ipure kit v2 (Diagenode, Liège, Belgium). For the H2Aub1/H2A.Z ChIP-seq experiment, the MicroPlex Library Preparation kit v2 (Illumina) was used for library preparation, and 50-bp single-end read sequencing was performed on the Hiseq 2000 platform (Illumina) at SciLifeLab (Uppsala, Sweden). For the H3K27me3 and REF6 ChIP-seq experiments, the Ovation Ultralow Sytem V2 (NuGEN, Redwood City, CA, USA) was used, and 150-bp paired-end sequencing was performed on the HiseqX platform at Novogene (Hong Kong). The ChIP-seq experiments were done using 2 biological replicates per IP, per genotype.

### Transcriptome data analysis

For our RNA-seq data and for the re-analysis of the RNA-seq data from [6], we employed cutadapt v. 1.18 [57] for adapter trimming and then mapped the reads to the *Arabidopsis* reference TAIR10 genome using the program tophat v2.1.1 [58], see Additional file 13: Table S12 for details on read numbers. Only reads mapping to nuclear chromosomes, reads with length > 25 nt, and reads with mapping quality > 30 were used in the subsequent analysis. Mapped reads were annotated and quantified using HTseq v0.11.1 [59]. DESeq2 [60] was used to analyse new (*ubp12*, *ubp13*, *ubp12/ubp13*) and previously published (*bmi1a bmi1b* [19], *ring1a ring1b* [19], *clf swn* [19], *emf1* [9]) raw transcript data, with differential analysis only for those genes with CPM > 1 in at least 2 replicates. Significantly deregulated genes were defined as having a |log2FC| ≥ 1 and a false discovery rate (FDR) adjusted *p* value (*p*_adj_) of < 0.05. PRC1 mutant transcriptome data from [20] was not re-analysed, and so here, the |lof2FC| cut-off of 4 was kept. When displaying the differential expression as boxplots, no prior filtering (on log2FC or *p* value) was performed and non-detected genes were assigned a log2FC value of 0 (except when stated otherwise). To produce the final graphs, we only displayed values associated with protein-coding nuclear genes (*N* = 27,206).

### H2Aub1, H2A.Z, H3K27me3, and REF6 ChIP-seq data analysis

FastQC [61] was used for quality control. Low-quality ends (phred of < 20) and adapter sequences were removed with Trimmomatic (version 0.38, [62]). Reads with low average quality were discarded too (phred < 25 for the H2Aub1/H2A.Z ChIP-seq experiment and < 28 for the H3K27me3 and REF6 experiments). For all experiments, reads were mapped to the *Arabidopsis* reference TAIR10 genome using bowtie2 (version 2.2.9 [63];), unpaired mode for the H2Aub1/H2A.Z experiment, and paired mode for the H3K27me3 and REF6 experiments. For the latter two, 50 bp was taken as the minimum distance between mates and 500 bp the maximum distance. After mapping, duplicated reads were removed. Details on read numbers can be found in Additional file 14: Table S13. For the H2Aub1/H2A.Z ChIP-seq experiment, fragment length was estimated with R function estimate.mean.fraglen, estimates can be found in Additional file 15: Table S14. For the H3K27me3 and REF6 ChIP-seq experiments, original fragments were reconstructed using information from both mates of a pair. Raw H2Aub1 and H3K27me3 data from a previous study was re-analysed in the same way as for our H2Aub1/H2A.Z ChIP-seq experiment. Gene annotation data was taken from the TAIR website (www.arabidopsis.org).

### H2Aub1, H2A.Z, H3K27me3, and REF6 peak calling

MACS2 [64] was used for H2Aub1, H2A.Z, and H3K27me3 peak calling, using H3 as a reference, and effective genome size of 119,481,543 bp. The broad mode was used with *q* = 0.05 and broad cut-off = 0.1. Peaks were considered real if they occurred in both replicates, in which case the peaks were merged. The presence of a mark on a gene requires the presence of at least one real peak in the first 1 kb of the gene body, see Additional file 6: Table S5 for the lists of genes defined by the presence of H2Aub1 and H3K27me3. H2Aub1 and H3K27me3 generated by Zhou et al. [6] have been re-analysed in the same way, with the exception that as control library, a common input library was used (no sample-specific input available). There is a 2–3-fold discrepancy in the number of H2Aub1-marked genes between our study and Zhou et al. We believe this is caused by the difference in plant material used (33 DAG rosettes vs 7 DAG seedlings), as the difference in peak annotation methods does not greatly affect the numbers, see Additional file 16: Table S15. For the REF6 at H2Aub1 peak summits, H2Aub1 peaks were called differently: reads from both replicates were merged, and peaks were called in narrow mode to obtain peak summits. The narrow mode was also used to call REF6 peaks; however, replicates were kept separate. Peaks from both replicates that were overlapping were merged; the rest of the peaks discarded.

### Identifying UBP12/13 targets

To identify UBP12/13 targets, we located regions with increased H2Aub1 in the *ubp12 ubp13* mutant compared to wt. To test for significant differential enrichment, we employed MAnorm [65]. MAnorm was run using MACS2 output directly with the following parameters: window size 1000 bp, number of simulations 100, *p* value cut-off 0.05, and m cut-off 0. The shift value was set as half the estimated fragment length (see Additional file 15: Table S14). From the output of the programme, peaks occurring in both replicates (overlapping with at least 1 bp) and with the same direction of change were considered real peaks and were merged, see Additional file 4: Table S3 for the lists of differential peaks. Peaks were annotated to protein-coding genes if they overlapped with the first 1 kb of the gene body. The final list of UBP12/13 targets contains genes with peaks of H2Aub1 increase, minus those genes that also contained a peak of H2Aub1 decrease (the latter only applied to 2 genes), see Additional file 5: Table S4 for a list of UBP12/13 target genes.

### H2Aub1, H2A.Z, H3K27me3, and REF6 scaling and normalizing

In order to display H2Aub1, H3K27me3, H2A.Z, and REF6 levels (as well as H3 or input controls) in metagene plots or genome browser images, we divided the genome into 50-bp bins and counted the number of fragments in each bin. We then divided the number of fragments per bin (+ 1) by the total base coverage (in billion bases). This has been done instead of scaling to the total number of fragments because of differing fragment lengths. A comparison of ChIP-seq replicates is shown in Additional file 1: Figure S16. After scaling, replicates were combined (by taking the mean), see Additional file 1: Figure S17 for a genome browser image of several genes with the scaled ChIP-seq tracks. To analyse differential H3K27me3 enrichment per gene, we performed a similar analysis, but instead of 50-bp bins, we counted the number of fragments within the first 1 kb, first 20%, and last 20% of gene bodies and applied the same scaling as before. After scaling, replicates were averaged, and scaled fragment counts from wild type were subtracted from mutant counts, see Additional file 17: Table S16 for differential H3K27me3 enrichment for all protein-coding nuclear genes. For the PRC1-dependent/independent metagene plots and for the correlation of H2aub1 and H3K27me3 with expression, we reanalysed previously published H2Aub1 and H3K27me3 data [6] in the same way our data was analysed.

### REF6 analyses

Regions hyper-methylated in *ref6* and regions bound by REF6 (REF6-HA) were obtained from [25] and were annotated to the first 1 kb of gene bodies using TAIR10. Genome-wide REF6 read counts and lists of CTCTGYTY motifs were also obtained from [25]. Lists of motif clusters were produced using the criteria of the original publication: at least 4 motifs in a 600-bp window. Overlapping clusters were merged. These analyses were repeated with our own REF6 ChIP-seq data. We also performed an analysis correlating H2Aub1 and REF6 level in 50-bp bins. For this analysis, we counted the number of motifs in 600-bp areas centred on the bins.

### Gene responsiveness analysis

Gene responsiveness scores were obtained from a previously published study [26]. We updated the annotation of the probes to TAIR10. Probes that were associated with multiple loci were not included in the analysis. The updated responsiveness list is presented in Additional file 10: Table S9.

### Gene Ontology analysis

To perform Gene Ontology analysis, we used the Panther Overrepresentation Test (released 2019-03-08), Annotation version ‘GO Ontology database Released 2019-02-02’, with the annotation dataset ‘GO biological process complete’ [66]. Fisher’s exact test was used with Bonferroni correction for multiple testing. The final results included only GO terms that passed the corrected *p* value of 0.05, which had at least 5 genes from the input list and that were overrepresented by genes from the input list.

### Graphical and statistical software

To produce most graphs, base R functions were used. For metagene plots, we used functions from the Deeptools linux package [55]. The program Sigmaplot (v. 14.0, Systat Software Inc.) was used to carry out Mann-Whitney *U* tests, hypergeometric tests and linear regression were performed in R, and ANCOVA was performed in IBM SPSS Statistics v. 24. Final figures were assembled and annotated (where necessary) using Inkscape.

## Supplementary information

**Additional file 1:Figure S1.** RNA-seq PCA highlights redundancy between *UBP12* and *UBP13.***Figure S2.** H2Aub1 western blot. **Figure S3.** UBP12/13 targets are enriched for upregulated genes. **Figure S4.** Differential expression in a PRC1 mutant. **Figure S5.** Gene deregulation in a PRC2 mutant. **Figure S6.** UBP12/13 preferentially target PRC1-dependent genes. **Figure S7.** H3K27me3 western blot. **Figure S8.** H2Aub1-only genes are over-enriched for genes hypermethylated in *ref6*. **Figure S9.** H2Aub1-only genes are over-enriched for REF6 targets. **Figure S10.** Motif clusters are not sufficient for REF6 binding. **Figure S11.** H2Aub1-only genes are over-enriched for REF6 targets. **Figure S12.** CTCTGYTY motif and H2Aub1 presence are positively associated with REF6. **Figure S13.** H2Aub1 is associated with gene responsiveness. **Figure S14.** UBP12/13 targets are enriched for H2A.Z and putative H2A.Zub1. **Figure S15.** H2A.Z presence does not affect expression change in UBP12/13 targets. **Figure S16.** Comparison of replicates for ChIP-seq. **Figure S17.** Genome browser image.

**Additional file 2:Table S1.** Genes deregulated in *ubp12*, *ubp13*, and *ubp12/13*.

**Additional file 3:Table S2.** GO analysis of genes upregulated in *ubp12*, *ubp13*, and *ubp12/13*.

**Additional file 4: Table S3.** Differential H2Aub1 peaks.

**Additional file 5: Table S4.** UBP12/13 target genes.

**Additional file 6: Table S5.** Gene groups defined by presence of H2Aub1 and H3K27me3 in wt.

**Additional file 7: Table S6.** Gene groups defined by presence of H2Aub1 and H3K27me3 in wt, and PRC1-dependency.

**Additional file 8: Table S7.** Gene groups defined by the presence of REF6.

**Additional file 9: Table S8.** GO analysis of H2Aub1-marked genes, and H3K27me3-marked genes.

**Additional file 10: Table S9.** Gene responsiveness.

**Additional file 11: Table S10.** Gene groups defined by the presence of H2A.Z.

**Additional file 12: Table S11.** Primers used in this study.

**Additional file 13: Table S12.** Numbers of reads for the RNA-seq experiment.

**Additional file 14: Table S13.** Numbers of reads for the ChIP-seq experiments.

**Additional file 15: Table S14.** Estimated mean of fragment lengths.

**Additional file 16: Table S15.** Peak annotation method comparision.

**Additional file 17: Table S16.** Differential H3K27me3 in *ubp12/13* vs wt.

**Additional file 18.** Review history.

## Data Availability

The datasets supporting the conclusions of this article are available in the Gene Expression Omnibus (GEO) repository, with accession number GSE131756 [67].
