# Supplementary material for: Removal of H2Aub1 by ubiquitin-specific proteases 12 and 13 is required for stable Polycomb-mediated gene repression in Arabidopsis
Source: Genome Biol. 2020 Jun 16;21:144. doi: 10.1186/s13059-020-02062-8 (PMC7296913; doi:10.1186/s13059-020-02062-8)
Supplement: Supplementary file 1 — Additional file 1:Figure S1. RNA-seq PCA highlights redundancy between UBP12 and UBP13.Figure S2. H2Aub1 western blot. Figure S3. UBP12/13 targets are enriched for upregulated genes. Figure S4. Differential expression in a PRC1 mutant. Figure S5. Gene deregulation in a PRC2 mutant. Figure S6. UBP12/13 preferentially target PRC1-dependent genes. Figure S7. H3K27me3 western blot. Figure S8. H2Aub1-only genes are over-enriched for genes hypermethylated in ref6. Figure S9. H2Aub1-only genes are over-enriched for REF6 targets. Figure S10. Motif clusters are not sufficient for REF6 binding. Figure S11. H2Aub1-only genes are over-enriched for REF6 targets. Figure S12. CTCTGYTY motif and H2Aub1 presence are positively associated with REF6. Figure S13. H2Aub1 is associated with gene responsiveness. Figure S14. UBP12/13 targets are enriched for H2A.Z and putative H2A.Zub1. Figure S15. H2A.Z presence does not affect expression change in UBP12/13 targets. Figure S16. Comparison of replicates for ChIP-seq. Figure S17. Genome browser image. [file 13059_2020_2062_MOESM1_ESM.docx]

**Additional File 1: Supplementary Figures.**

**
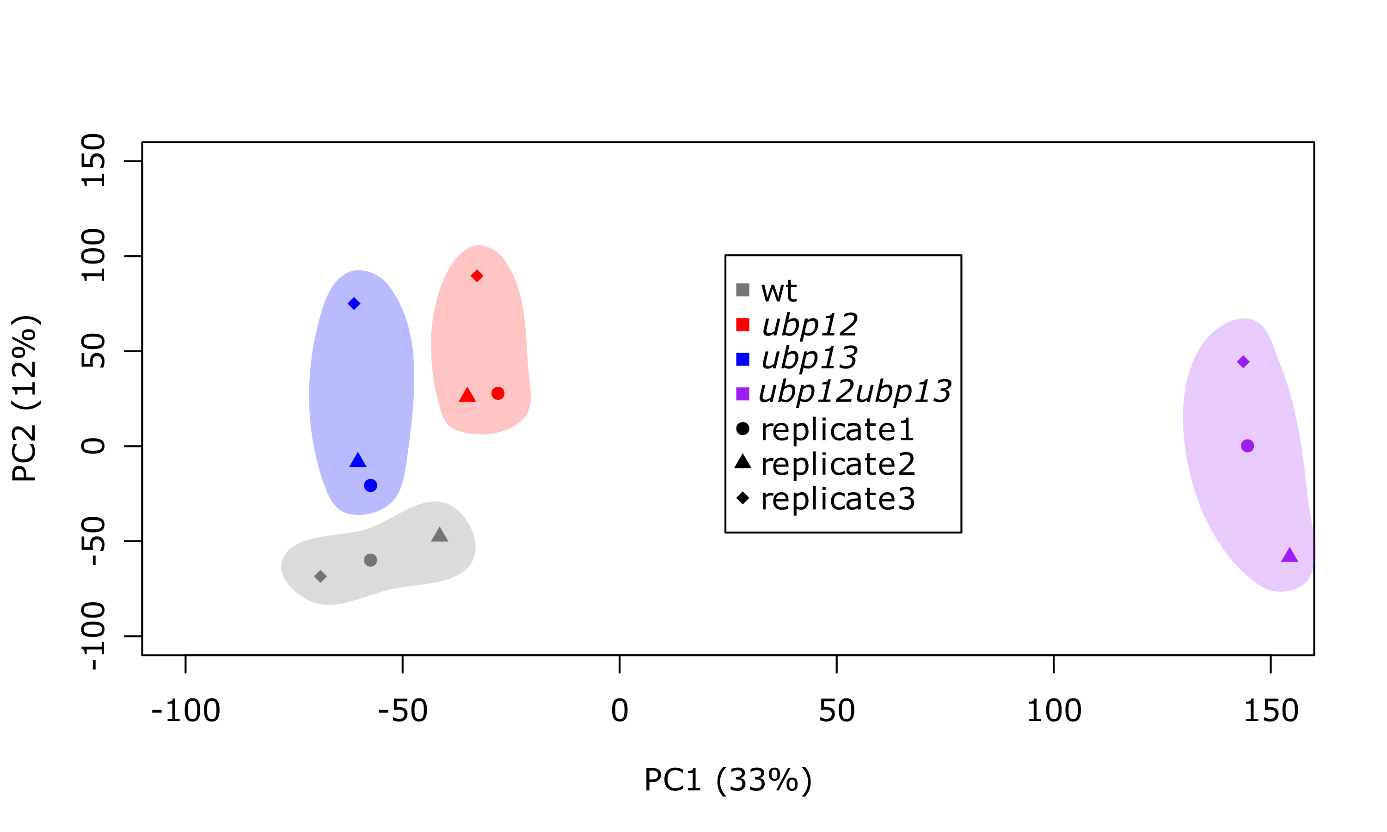
**

**Figure S1. RNA-seq PCA highlights redundancy between *UBP12* and *UBP13*.** PCA plot of RNA-seq rpkm values of each of three replicates for each of the four genotypes (wt, *ubp12*, *ubp13*, *ubp12/13*).


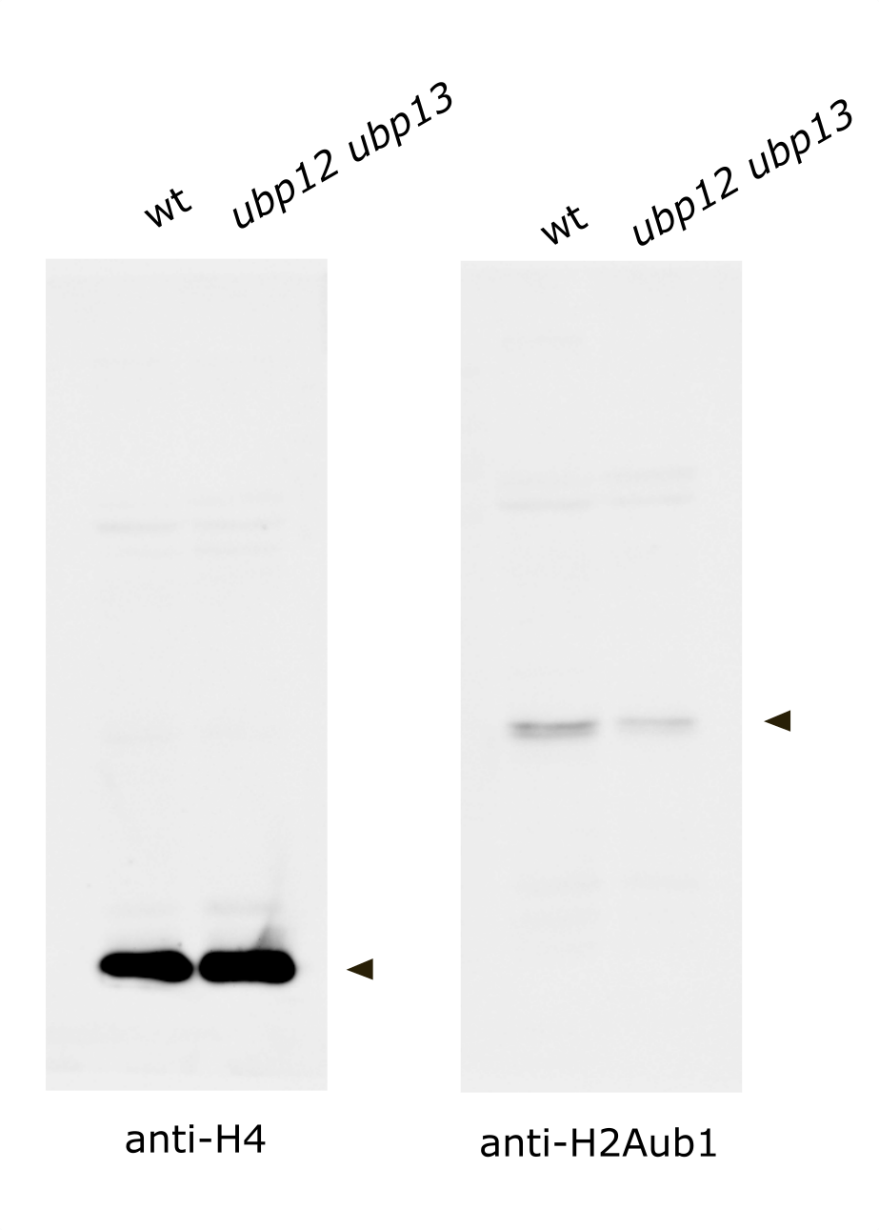


**Figure S2. H2Aub1 western blot.** Western blots of histone extracts of whole seedlings of wild-type and *ubp12-2w ubp13-3*, probed with anti-H4 (left), or anti-H2aub1 (right). Equal amount of plant material were used for the different samples. The arrows indicate the location of the H4 and H2Aub1 bands.

**
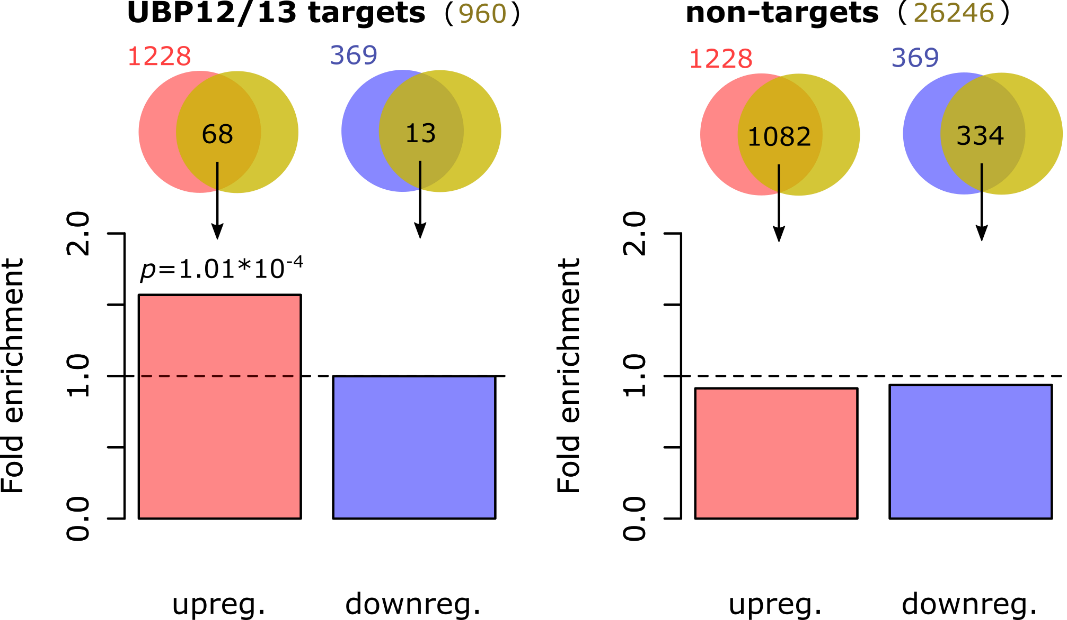
**

**Figure S3. UBP12/13 targets are enriched for upregulated genes.** Overlap of gene lists (actual/ expected based on chance) of UBP12/13 targets (right) or non-targets (left), and genes significantly upregulated (log2FC >1, *p*_adj_<0.05) or genes downregulated (log2FC<-1, *p*_adj_<0.05) in *ubp12/13*. The dashed line indicates the expected overlap based on chance. *p*-values are obtained from a hypergeometric test for significant overenrichment.


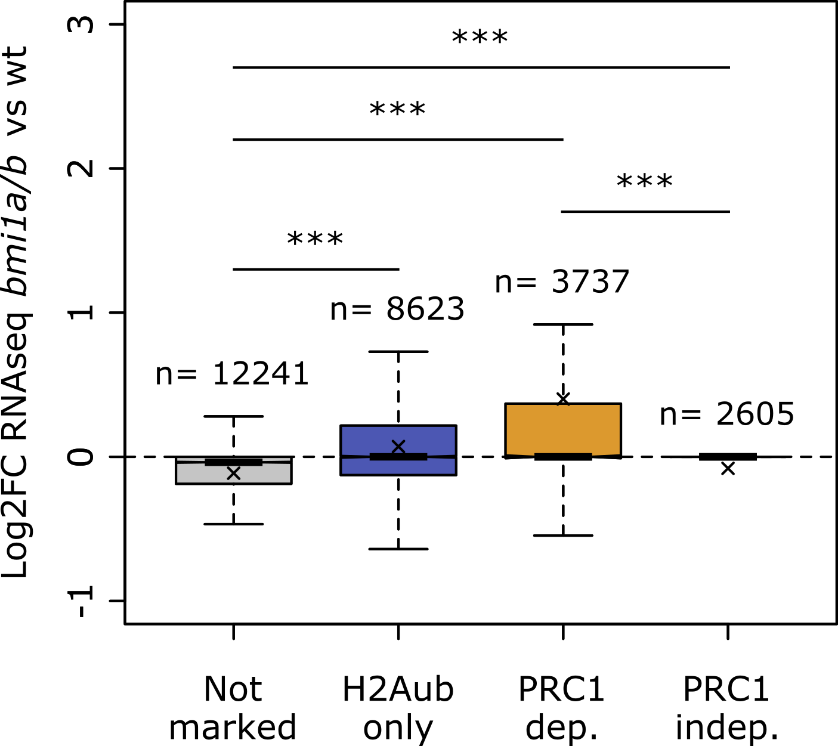


**Figure S4. Differential expression in a PRC1 mutant.** Differential expression in *bmi1a/b* compared to wt for 4 categories defined by the presence of H2Aub1 and H3K27me3 and the dependency of the H3K27me3 mark on PRC1. Mann-Whitney *U* test were performed to test for significant differences in distributions of data, ns *p*≥0.05, * *p*<0.05, ** *p*<0.01,*** *p*<0.001; Bonferroni correction was applied with m=4. Based on re-analyzed previously published data (ChIP-seq[1], RNAseq[2]).


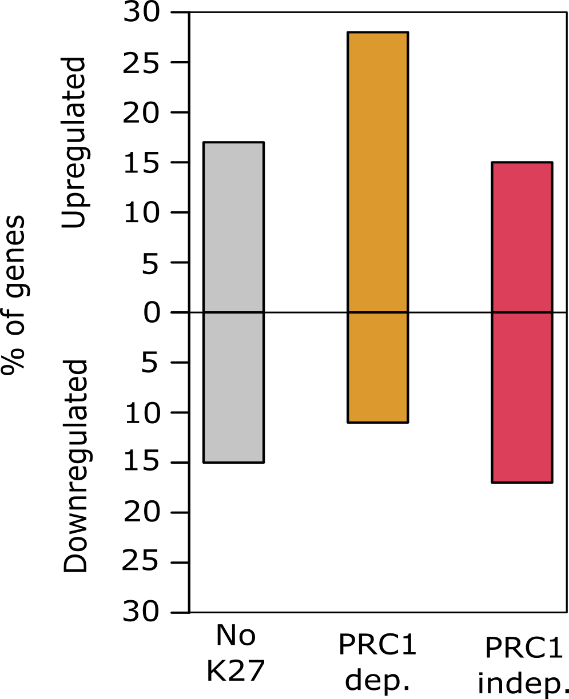


**Figure S5. Gene deregulation in a PRC2 mutant.** Percentage of genes upregulated and downregulated in a prc2 double mutant (*clf swn*) among gene categories defined by the presence of H3K27me3 and dependency of H3K27me3 on PRC1. Significant differential expression required a |log2FC| >1 and *p*_adj_<0.05. Based on re-analyzed previously published data [2].


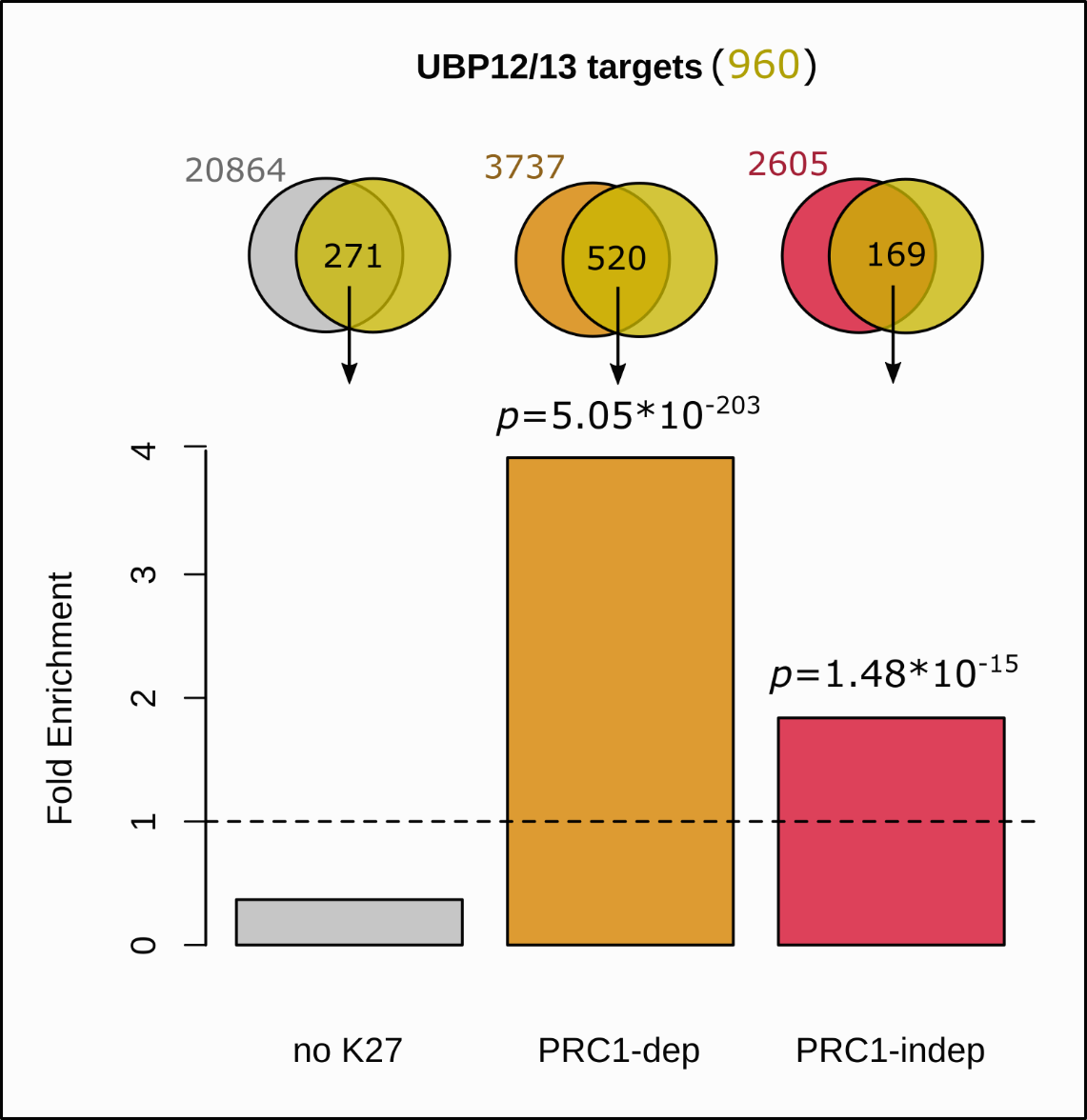


**Figure S6.** **UBP12/13 preferentially target PRC1-dependent genes.** Fold enrichment of the overlap of UBP12/13 target genes (960 genes), and genes defined by presence of H3K27me3, and whether the H3K27me3 (K27) on those genes is dependent on PRC1 activity (dep=dependent, indep= independent). The dashed line indicates the expected overlap based on chance alone. *p*-values are obtained from a one-tailed hypergeometric test for overenrichment.


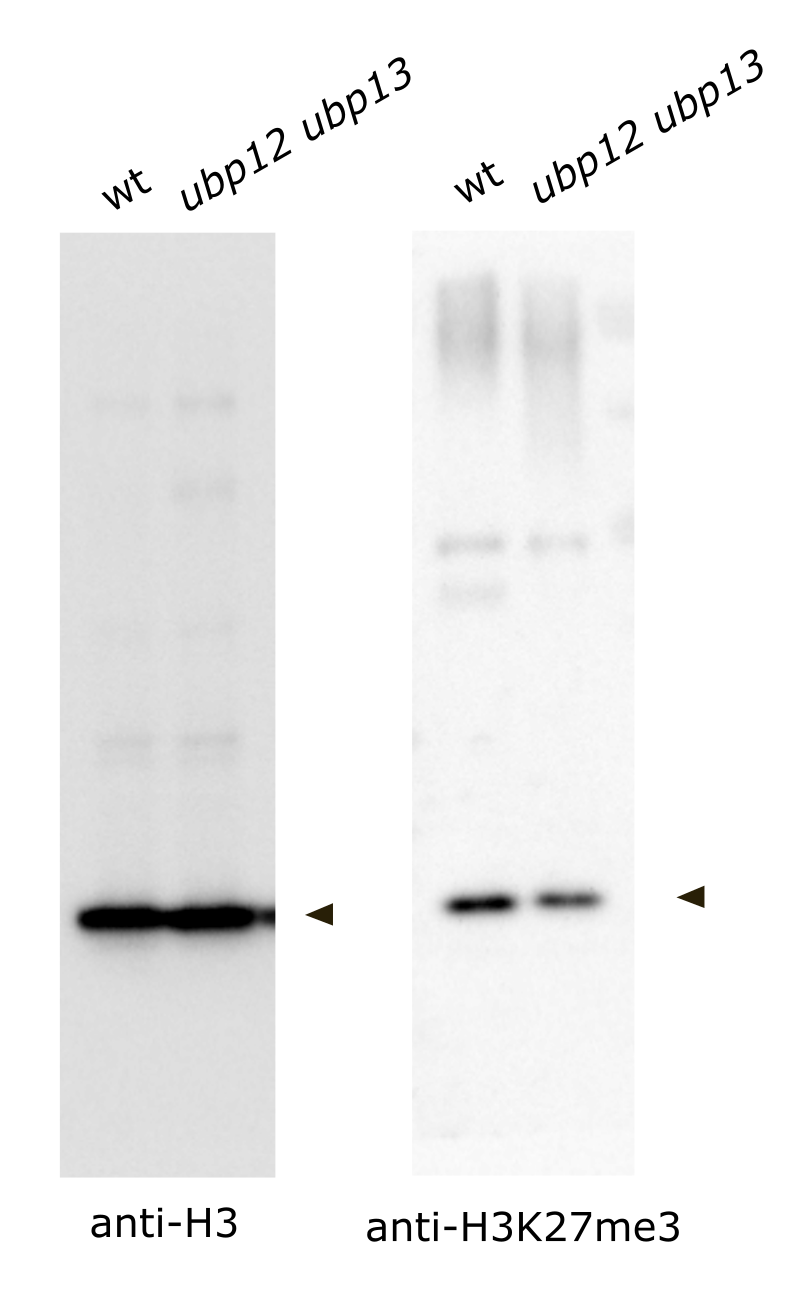


**Figure S7. H3K27me3 western blot.** Western blots of histone extracts of 33 DAG rosettes of wild-type and *ubp12-1 ubp13-3*, probed with anti-H3 (left) or anti-H3K27me3 (right). Equal amounts of plant material was used for the different samples. The arrows indicate the location of the H3 and H3K27me3 bands.


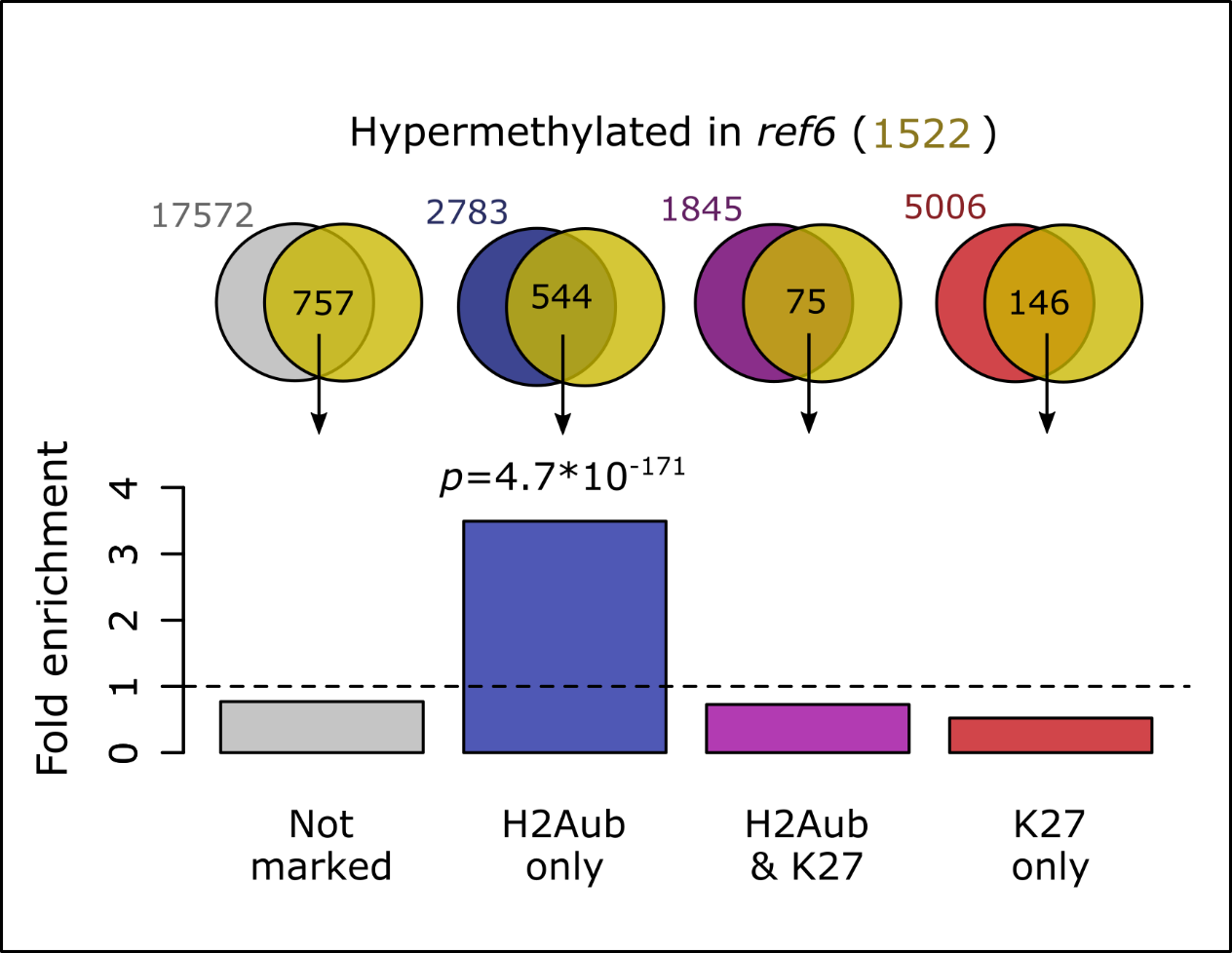


**Figure S8. H2Aub1-only genes are over-enriched for genes hypermethylated in *ref6*.** Fold enrichment of the overlap of genes gaining H3K27me3 in *ref6* (1552 genes), and genes defined by presence of H2Aub1 (H2Aub) and H3K27me3 (K27). The dashed line indicates the expected overlap based on chance alone. *p*-values are obtained from a one-tailed hypergeometric test for overenrichment. Based on re-analyzed published data [3].


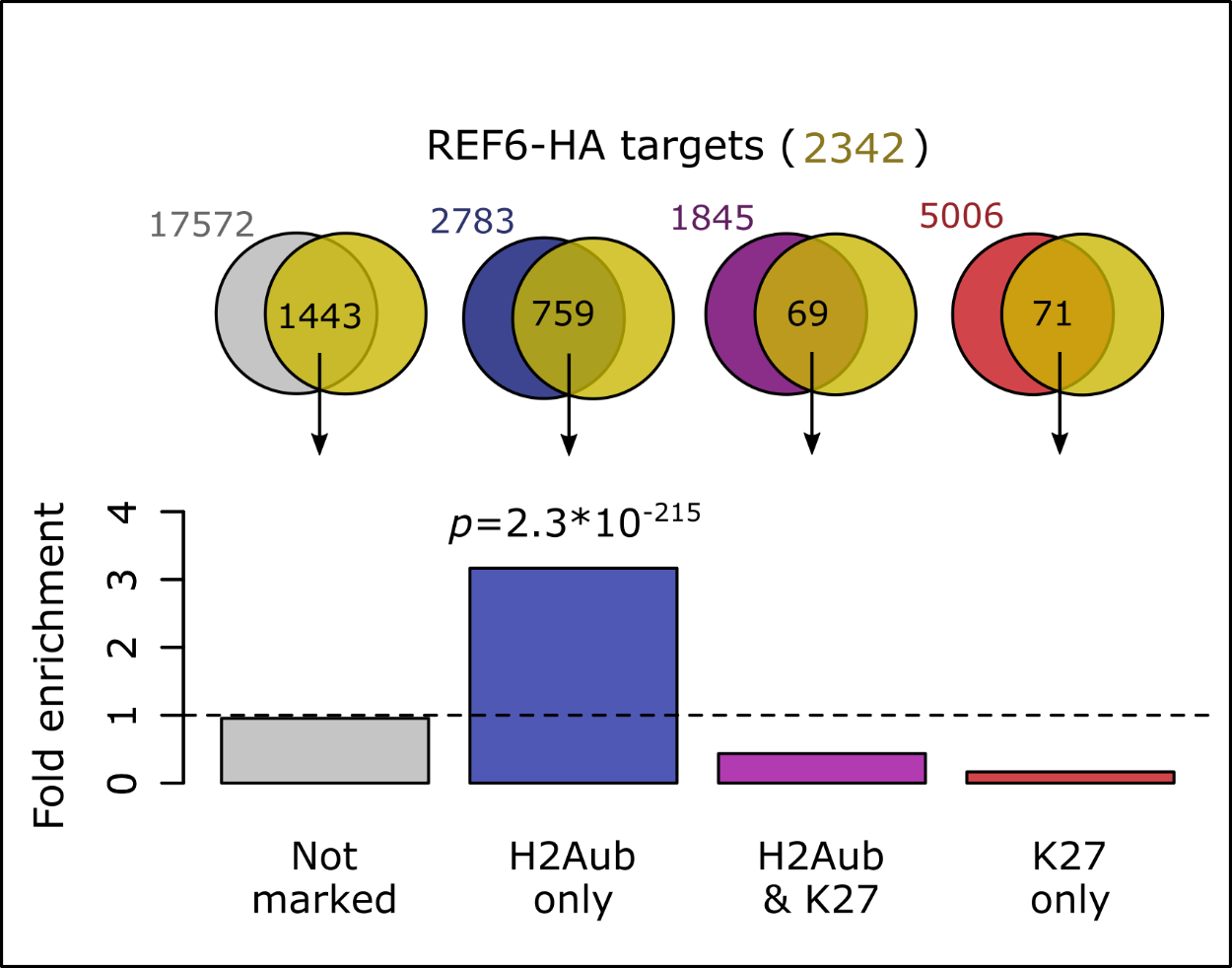


**Figure S9. H2Aub1-only genes are over-enriched for REF6 targets.** Fold enrichment of the overlap of genes with significant REF6 peaks (2342 genes), and genes defined by presence of H2Aub1 (H2Aub) and H3K27me3 (K27). The dashed line indicates the expected overlap based on chance alone. *p*-values are obtained from a one-tailed hypergeometric test for overenrichment. Based on re-analyzed published data [3].

**
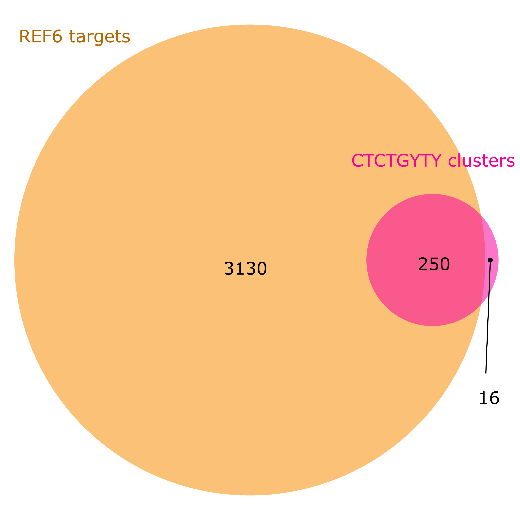
**

**Figure S10. Motif clusters are not sufficient for REF6 binding.** “REF6 targets” indicates all areas in the genome with significant REF6 peaks as determined by [3], and “CTCTGYTY clusters” indicates all areas in the genome that have at least 4 CTCTGYTY motifs in a 600 bp window. Based on re-analyzed published data [3].


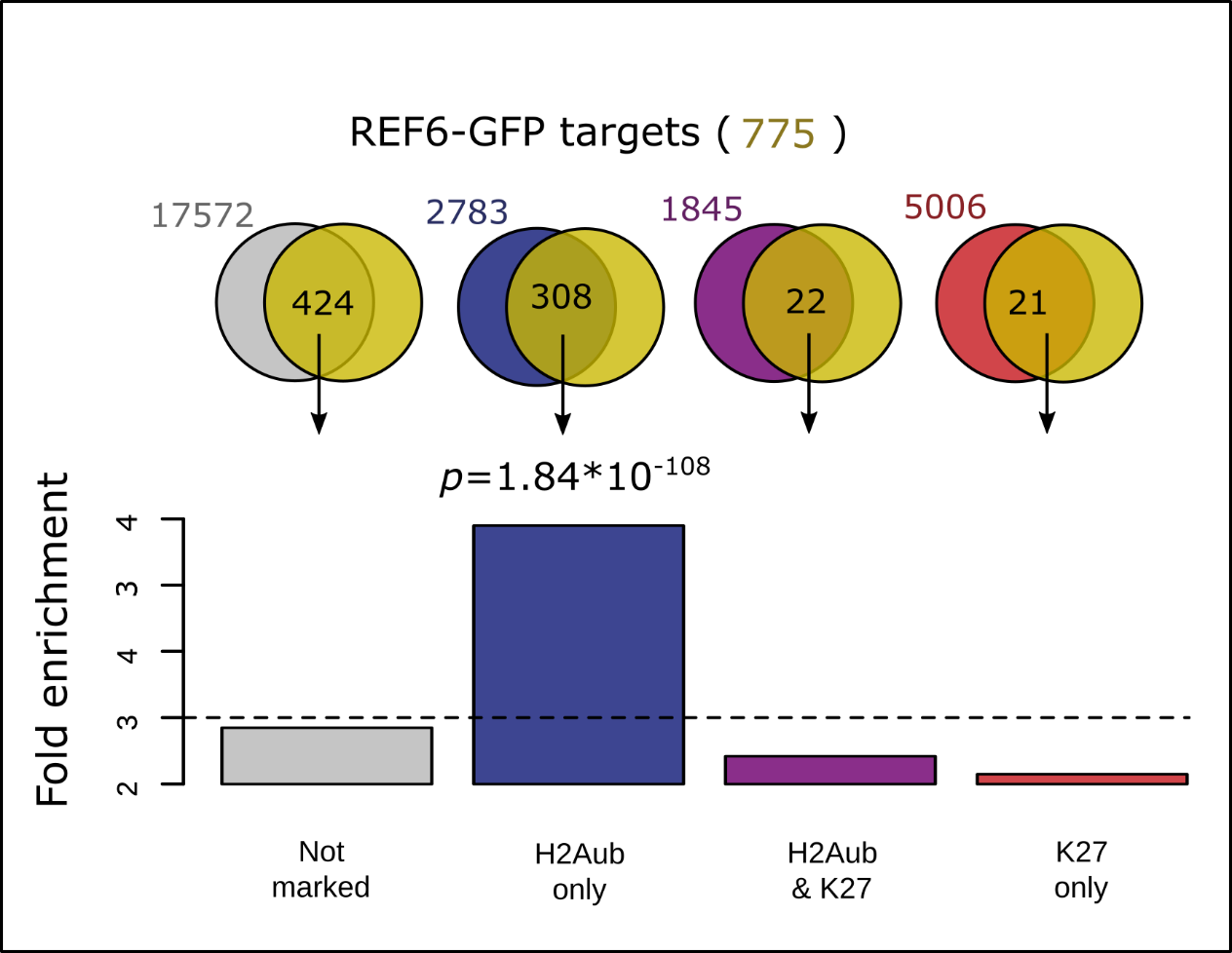


**Figure S11. H2Aub1-only genes are over-enriched for REF6 targets.** Fold enrichment of the overlap of genes with significant REF6 peaks (775 genes), and genes defined by presence of H2Aub1 (H2Aub) and H3K27me3 (K27). The dashed line indicates the expected overlap based on chance alone. *p*-values are obtained from a one-tailed hypergeometric test for overenrichment.


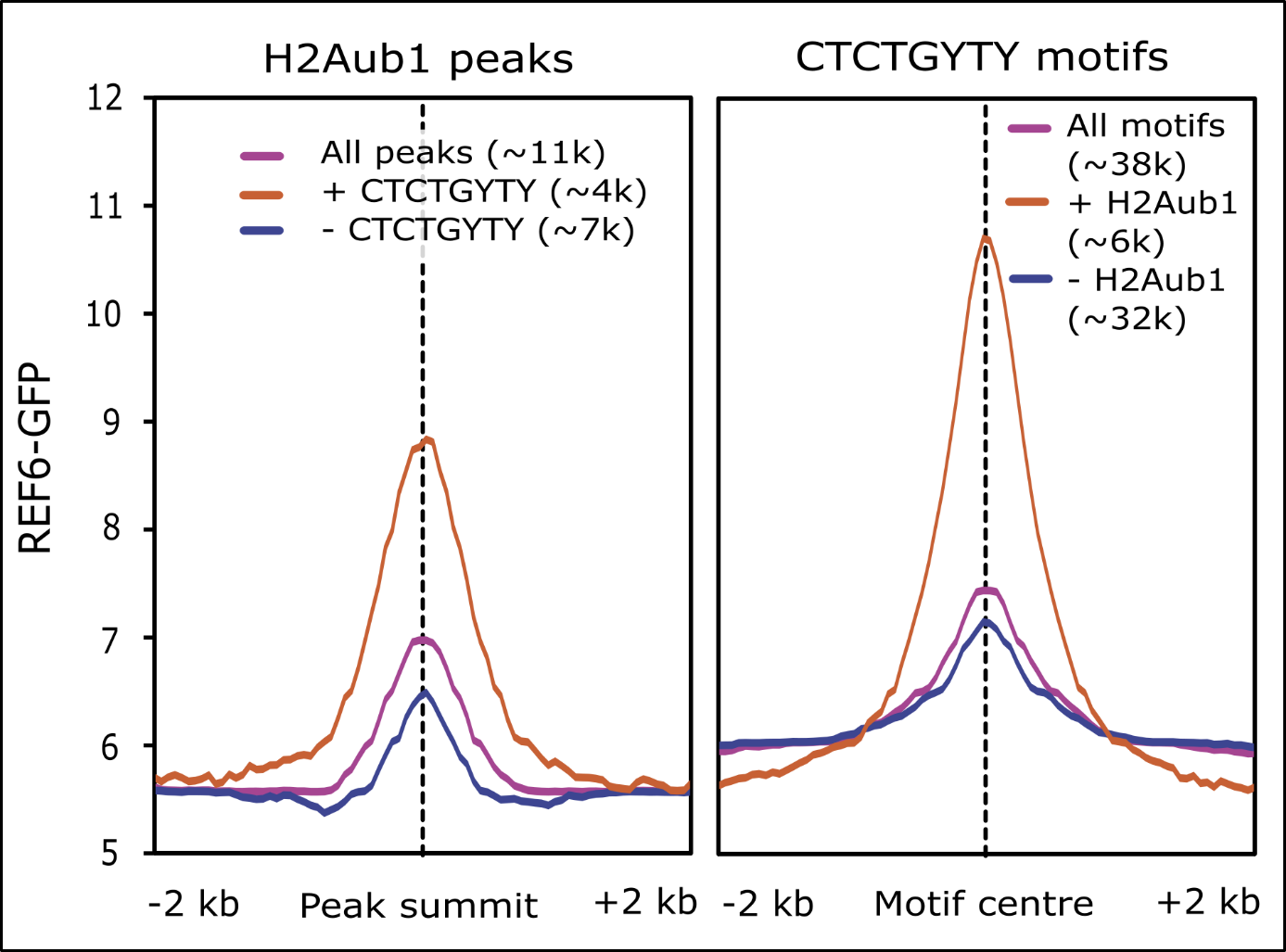


**Figure S12. CTCTGYTY motif and H2Aub1 presence are positively associated with REF6.** REF6 enrichment (median of the coverage-scaled fragment count) at narrow H2Aub1 peaks (left) and at CTCTGYTY motifs (right). Depicted in the left panel are all significant H2Aub1 peaks (purple), all peaks with at least one CTCTGYTY motif (red), and all peaks that do not possess at least one CTCTGYTY motif (blue). Depicted in the right panel are all CTCTGYTY motifs (purple), all motifs with H2Aub1 (red), and all motifs without H2Aub1 (blue).

**
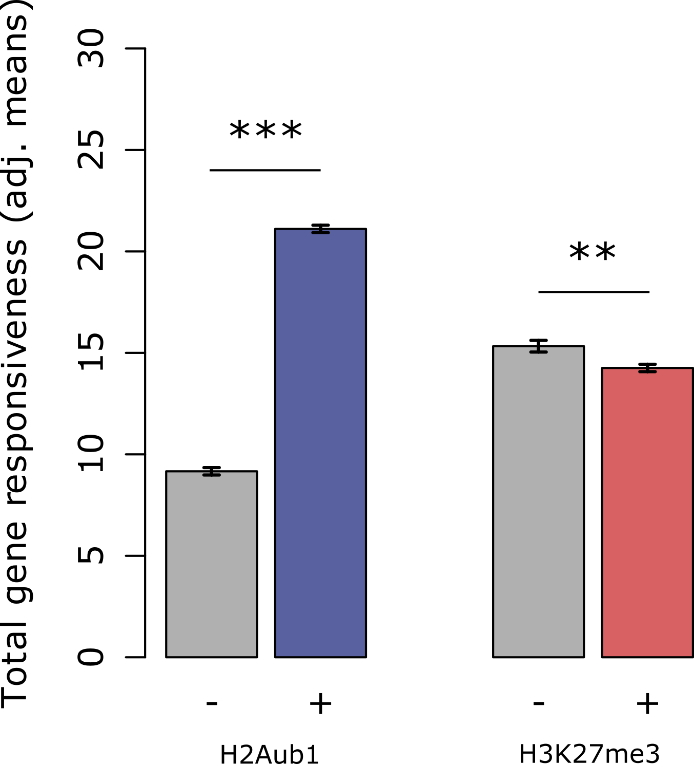
**

**Figure S13. H2Aub1 is associated with gene responsiveness.** Total gene responsiveness as defined by [4], i.e. a score representing in how many different conditions the genes change their expression compared to the control condition (e.g. a low score indicates a stable expression across conditions), in absence (-) or presence (+) of H2Aub1 or H3K72me3, correcting for the presence of the other mark (estimated marginal means). Based on re-analyzed previously published data [1, 4]. Test for significance by ANCOVA post-hoc test as implemented by SPSS, ns *p*≥0.05, * *p*<0.05, ** *p*<0.01,*** *p*<0.001. Bonferroni correction applied with m=2.


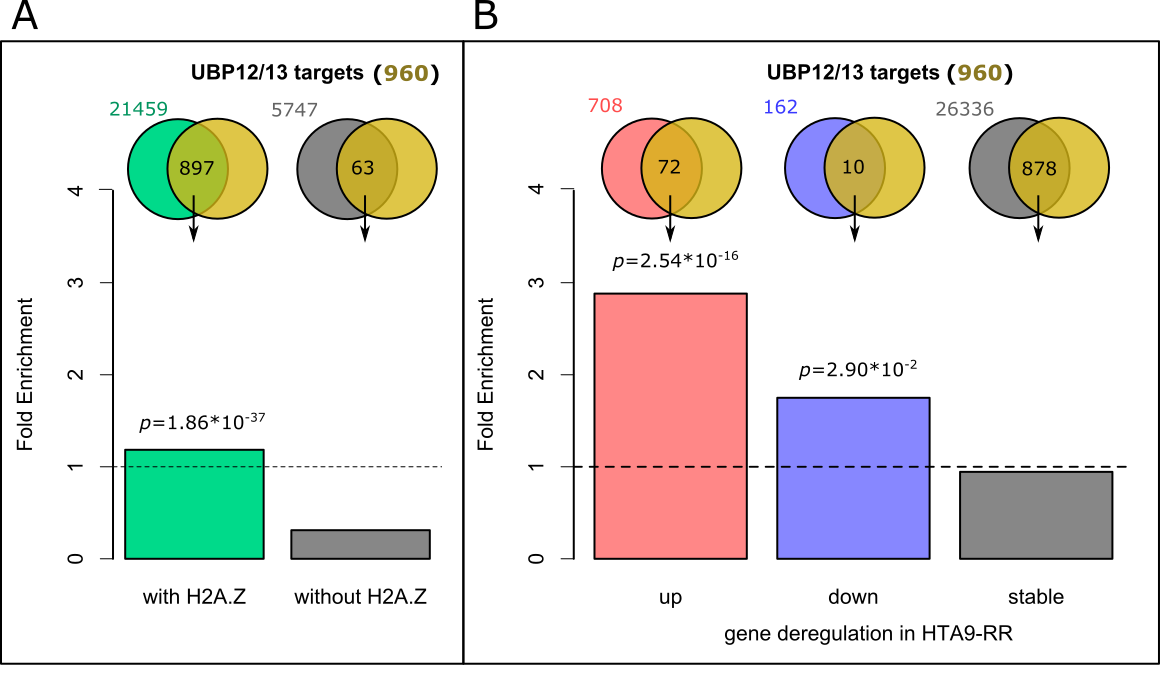


**Figure S14. UBP12/13 targets are enriched for H2A.Z and putative H2A.Zub1.** **A)** Fold enrichment of the overlap of UBP12/13 target genes (960) and genes with (21559) or without (5747) HTA9. **B)** Fold enrichment of the overlap of UBP12/13 target genes with genes upregulated (708), downregulated (162) or unchanged (26336) in *hta9* HTA9-RR versus wild type (minus those genes deregulated in *hta9* HTA9-N versus wild type). *p*-values are obtained from a one-tailed hypergeometric test for overenrichment.


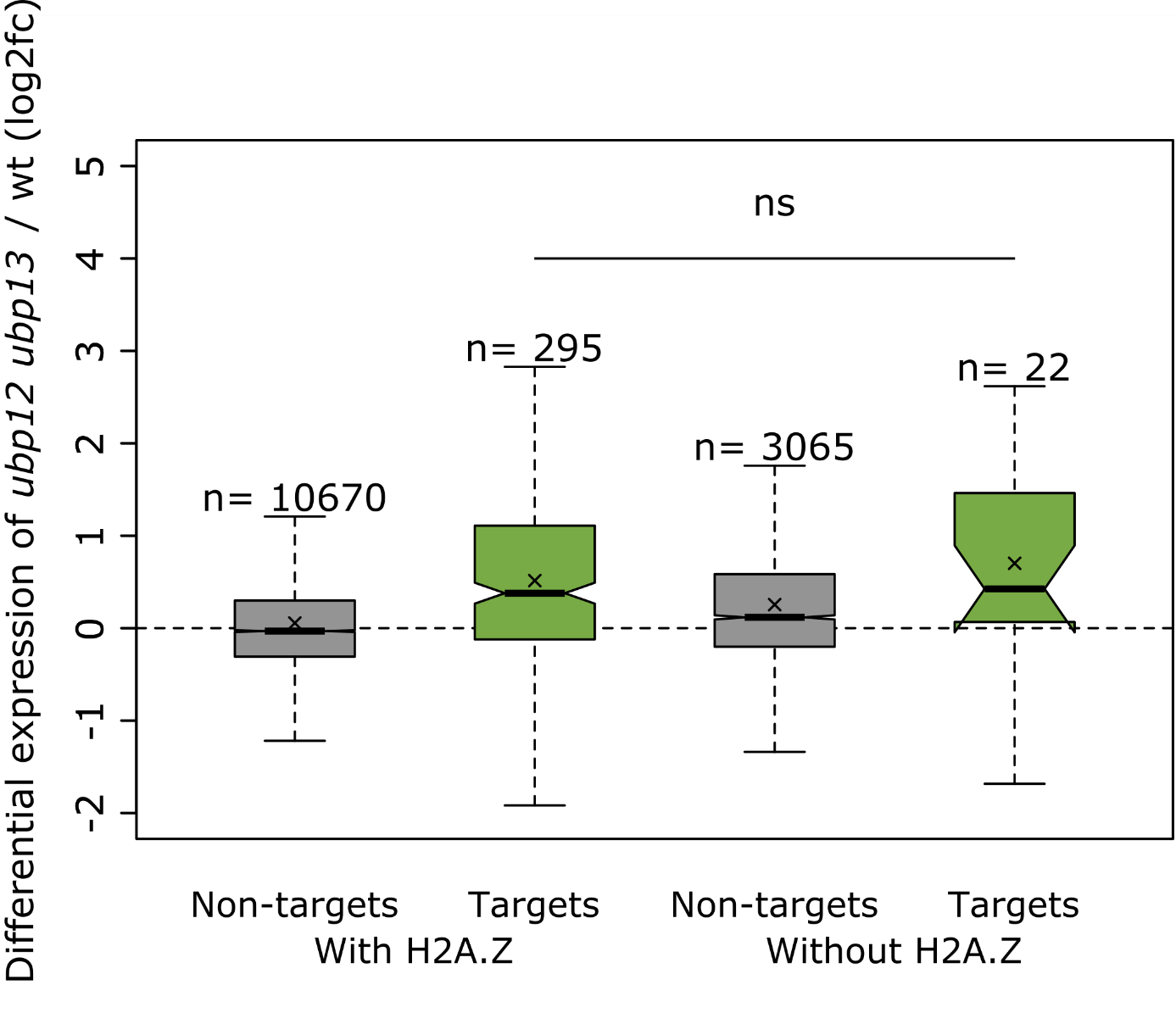


**Figure S15. H2A.Z presence does not affect expression change in UBP12/13 targets.** Boxplots showing differential expression of all expressed UBP12/13 targets (green), and all expressed other protein-coding genes (grey), either with (left) or without (right) H2A.Z. Test for significance was done by Mann-Whitney *U* test, ns *p*≥0.05, * *p*<0.05, ** *p*<0.01,*** *p*<0.001.


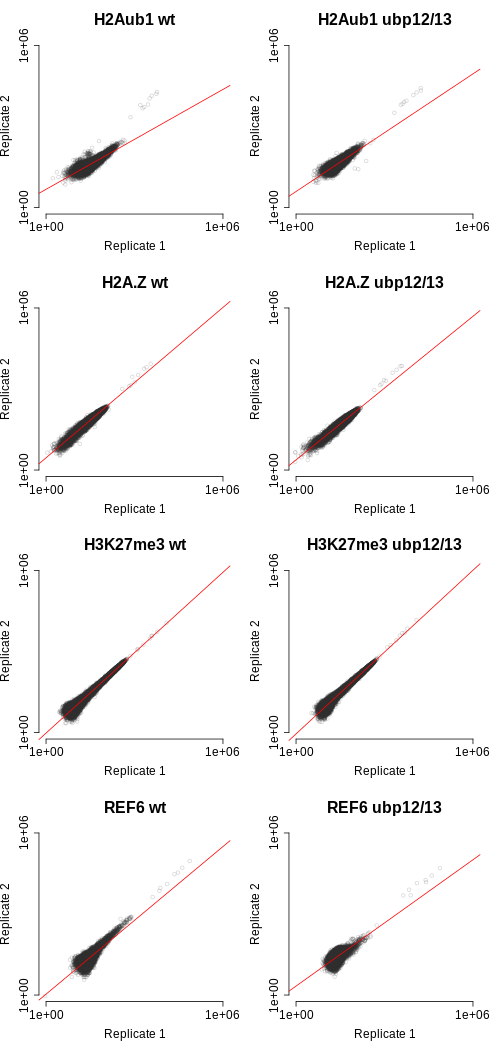


**Figure S16. Comparison of replicates for ChIP-seq.** Each point represents the total coverage-scaled fragment count across the first 1 kb of the gene body of a gene, the X coordinate the count in replicate 1 and the Y coordinate the count in replicate 2. Only protein-coding genes are included in this analysis. Left-hand panes are for wt (Col-0), and right-hand panes for the *ubp12/13* mutant. Lines are linear regression lines. X and Y axes are logarithmic (Log10). Statistics: H2Aub1 wt (m=0.62, R^2^=0.73, *p*<2.2E-16), H2AUb1 *ubp12/13* (m=0.73, R^2^=0.74, *p*<2.2E-16), H2A.Z wt (m=0.93, R^2^=0.94, *p*<2.2E-16), H2A.Z *ubp12/13* (m=0.89, R^2^=0.93, *p*<2.2E-16), H3K27me3 wt (m=0.99, R^2^=0.97, *p*<2.2E-16), H3K27me3 *ubp12/13* (m=1.00, R^2^=0.96, *p*<2.2E-16), REF6 wt (m=0.91, R^2^=0.63, *p*<2.2E-16), REF6 *ubp12/13* (m=0.78, R^2^=0.44, *p*<2.2E-16).


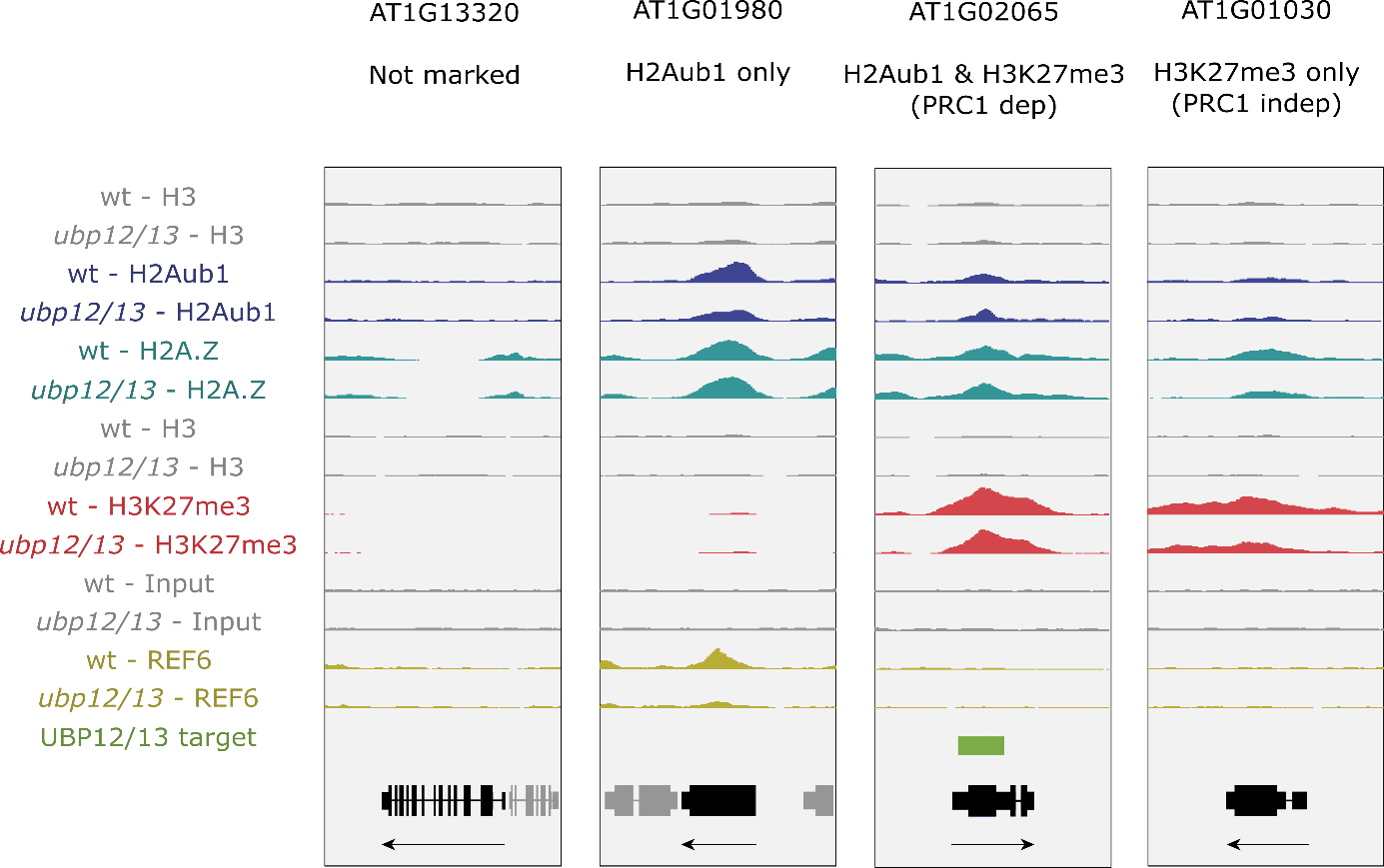


**Figure S17. Genome browser image.** Shown are representative genes in the 4 categories based on the presence of H2Aub1 and H3K27me3. The H2Aub1 & H3K27me3 gene here also happens to be a PRC1 dependent gene, and the H3K27me3-only gene happens to be PRC1 independent. Most tracks show number of fragments per 50bp bins scaled for total coverage for either wt or *ubp12/13* mutant, with the mean taken of the two replicates. The exception is the last track which shows which areas according to MANorm have increased H2Aub1 in *ubp12/13* in both replicates(and hence are called UBP12/13 targets). The fragment count range to display was set at the same level for the IP of interest as for the relevant (H3 or input) control. Below the tracks in black is the gene model that was focused on, with the arrow indicating the direction of transcription. Nearby genes are colored grey.

**Supplementary figure references**

1. Zhou Y, Romero-Campero FJ, Gomez-Zambrano A, Turck F, Calonje M: H2A monoubiquitination in Arabidopsis thaliana is generally independent of LHP1 and PRC2 activity**.** *Genome Biol* 2017, 18:69.

2. Wang H, Liu C, Cheng J, Liu J, Zhang L, He C, Shen WH, Jin H, Xu L, Zhang Y: Arabidopsis Flower and Embryo Developmental Genes are Repressed in Seedlings by Different Combinations of Polycomb Group Proteins in Association with Distinct Sets of Cis-regulatory Elements**.** *PLoS Genet* 2016, 12:e1005771.

3. Cui X, Lu F, Qiu Q, Zhou B, Gu L, Zhang S, Kang Y, Cui X, Ma X, Yao Q, et al: REF6 recognizes a specific DNA sequence to demethylate H3K27me3 and regulate organ boundary formation in Arabidopsis**.** *Nature Genetics* 2016, 48:694-699.

4. Aceituno FF, Moseyko N, Rhee SY, Gutierrez RA: The rules of gene expression in plants: organ identity and gene body methylation are key factors for regulation of gene expression in Arabidopsis thaliana**.** *BMC Genomics* 2008, 9:438.
