# Supplementary material for: Removal of H2Aub1 by ubiquitin-specific proteases 12 and 13 is required for stable Polycomb-mediated gene repression in Arabidopsis
Source: Genome Biol. 2020 Jun 16;21:144. doi: 10.1186/s13059-020-02062-8 (PMC7296913; doi:10.1186/s13059-020-02062-8)
Supplement: Supplementary file 18 — Additional file 18. Review history. [file 13059_2020_2062_MOESM18_ESM.docx]

Review History

**First round of review**

**Reviewer 1**

**Are you able to assess all statistics in the manuscript, including the appropriateness of statistical tests used?** No, I do not feel adequately qualified to assess the statistics.

**Comments to author:**

In this submission, Kralemann et al described that UBP12/13-mediated H2A deubiquitination prevented loss of H3K27me3 and played a critical role in stable PRC2-mediated repression in the Arabidopsis genome. Most of the data analyses in this study are well performed. Some concerns are outlined below.
Major criticisms:
1. The authors classified all of the loci gained H2Aub1 in ubp12 ubp13 (vs WT) as direct UBP12/13 targets, and all subsequent analyses used this group of genes, which is not appropriate as some of these genes are expected not to be direct targets of UBP12/13. To obtain the direct targets, ChIP-seq using anti-UBP12/13 (or epitope-tagged UBP12/13) needs to be performed.
2. The proposed model is too speculative.

Minor comments:
1. In previous study, Zhou et al. reported that H2Aub1 is widespread in the Arabidopsis genome (over 14,000 genes with this mark; Genome Biol. 18:69). It appears that only around 4,000 loci were identified with H2Aub1 in this study. Please comment on this discrepancy.
2. Line 169 (p7): please specify the four PRC1 mutants and add a brief description on functions of relevant genes.
3. Lines 183-185: "This suggests that PRC1-independent H3K27me3-marked genes are already repressed before PRC2 recruitment, differing from PRC1-dependent genes that require PRC2 for repression." This sentence does not make much sense. There should be a PRC2-recruitment mechanism in all H3K27me3-marked loci.
4. Lines 290-291: "Nevertheless, our study concentrated on genes gaining H2Aub1 in ubp12 ubp13 mutants, which is a consequence of UBP12/13 acting as histone deubiquitinase." Some of these genes gained H2Aub1 are expected not to be direct targets of UBP12/13.

**Reviewer 2**

**Are you able to assess all statistics in the manuscript, including the appropriateness of statistical tests used?** No, I do not feel adequately qualified to assess the statistics.

**Comments to author:**

In this manuscript, Kralemann et al investigate the role played by histone H2A monoubiquitination and removal of this H2Aub PRC1 mark by the UBP12 and UBP13 de-ubiquitinases, and their relationship with PRC2-mediated gene regulation by Polycomb activities. They mostly performed two series of RNA-seq and ChIP-seq experiments (of the H2Aub and of H3K27me3 histone marks) in wild-type and in double ubp12ubp13 Arabidopsis thaliana mutant plants, and compared the datasets. This allowed the authors to provide an original and interesting model of how these UBPs could influence transcription both positively or negatively, and their link with H3K27me3 deposition or spreading. More specifically, the proposal that H2Aub removal by UBP12/13 makes part of a "protective" mechanism against H3K27me3 removal by REF6 or by other de-methylases on some loci is very interesting.

This study follows the previous identification of UBP12 and UBP13 as the main histone H2Aub de-ubiquitinases in Arabidopsis by the same team. It also follows another study recently published on this topic (Gómez-Zambrano et al., Nature Com., June 2019), which unveiled a functional dichotomy in histone H2A.Z relationship with transcription, depending on whether this histone variant is mono-ubiquitinated by PRC1 or not. This recent concurrent publication does not address UBP12/UBP13 function and, in my opinion, only marginally overlaps with the submitted manuscript. Hence, I have considered this manuscript has having sufficient originality for the Genome Biology readership. Taken together, despite addressing very important aspects of the gene regulatory mechanisms in plants, in my opinion this study suffers from several lack of evidence supporting the proposed model and, to some extent, some the claims.

Major issues:
- The recent study from the Calonje lab, (Gómez-Zambrano et al, 2019) should be cited and used in future revised versions of this manuscript. It has now been released publicly for several weeks and, in particular, the conclusion that PRC2 acts both in PRC1-dependent and independent ways is also presented there. Also, the fact that H2Aub ChIP-seq generated and presented here might correspond to a mix of canonical H2A and H2A.Z variants should be discussed, as both sub-populations of H2Aub could behave very differently with regard to PRC2 activity/spreading and with UBP12/UBP13 activities. Hence, it would be interesting to integrate some of the data published by Gómez-Zambrano et al in additional comparisons and analyses to determine how much of the main conclusions of the manuscript would hold true for de-ubiquitination of canonical H2A versus H2A.Z variant.

- From the bioinformatics analyses of transcriptomic and chromatin profiles in wild-type and ubp12ubp13 plants, three important mechanistic models are proposed, which unfortunately are not supported by experimental evidences at this stage.
    Firstly, on line 136, a list of UBP12/13 target loci is defined, which relies on a combination of transcriptomic analyses and of H2Aub profile comparisons between wild-type and ubp12ubp13 plants. Considering the importance of this statement, and the extensive usage of this list in the manuscript for many analyses, it would be important to determine actual "true" direct targets by ChIP-seq of one of these two redundant proteins.
    Secondly, it is concluded on line 185 that "PRC1 recruits PRC2 to active genes to mediate their repression". This conclusion makes sense but requires a real mechanistic assessment, first in term of PRC2 recruitment (for example by ChIP analysis of PRC2 proteins to PRC1-dependent loci) and by a functional assay. From the data presented, this conclusion can only presented as a working model.
    Thirdly, the proposal that "H2Aub aids in the recruitment of the H3K27me3 demethylase REF6 needs additional support, in particular because of the great novelty of the model. For example, REF6-HA can be used for ChIP analyses on such loci in plants impaired in H2A deposition (e.g., bmi mutant plants) or H2Aub removal (ubp12/13 mutant plants).

- I missed the rationale for the description of decreased H2Aub levels in ubp12ubp13 mutant plants as compared to wild-type plants, as the exact opposite would be expected.

- ChIP-seq data showing low H3K27me3 levels in ubp12ubp13 mutant plants should be supported by western-blots, as done for the high levels of H2Aub. In the absence of internal standards for normalization, ChIP-seq is only partially quantitative. Furthermore, this complementary western-blot would exemplify the antagonistic relationships between H3K27me3 and H2Aub levels in this mutant.

-  I found no way to examine the ChIP-seq profiles generated in this study, as GEO accession requires a token. A token might have been generated for the reviewers but I did not found it in the data available. The anti-H2Aub antibody is notoriously difficult, and one would like to inspect the quality of the ChIP-seq presented here by looking at specific loci instead of relying on meta-gene analyses that smoothen even the dirtiest ChIP profiles.


Minor issues:

- On line 60, H2A mono-ubiquitination is indicated as H2AK121ub. However, there are multiple H2A variants and the numbering of the lysine residue targeted by PRC1 can change depending on each histone H2A isoform.

- In the Methods, there is no indication on the number of biological replicates for RNA-seq and ChIP-seq experiments. From the Supplementary figures, I understand 2 replicates were used in each RNA-seq or ChIP-seq experiment. Please detail this and how both replicates where integrated for all bioinformatics analyses.

- From the Methods I understand that RNA-seq have been performed on Arabidopsis seedlings whereas ChIP-seq have been performed on rosette leaves. The rationale for this choice is surprising. This information should be stated in the main text and eventually discussed.

- The individual panels of the figures are poorly cited in the text, making the readership more difficult. Also, the numbering of Figure 3 in the text should be corrected (3D cited instead of 3B) and in the legends (C, 1552 genes and not E, 1552 genes; D, 2342 genes and not F, 2342 genes).

- Related to the GEO datasets accessibility, in most instances, the definition of PRC1-dep, PRC1-indep, or other gene/peak types could be exemplified by representative browser profiles in the Figures, helping the reader to visually inspect how these different contexts look like.

**Authors Response**

**Point-by-point responses to the reviewers’ comments:**

Reviewer #1

1.The authors classified all of the loci gained H2Aub1 in ubp12 ubp13 (vs WT) as direct UBP12/13 targets, and all subsequent analyses used this group of genes, which is not appropriate as some of these genes are expected not to be direct targets of UBP12/13. To obtain the direct targets, ChIP-seq using anti-UBP12/13 (or epitope-tagged UBP12/13) needs to be performed.

a.We followed three approaches to obtain direct UBP12/13 targets, but unfortunately none of them *were successful. We generated lines with C-and N-terminally tagged UBP12 under control of the 35S promoter, but this construct failed to complement the mutant phenotype. We then asked for a published UBP12 antibody (Cui et al. 2013, Plant Physiology), which however was no longer available. We lastly asked for a published pUBP12:UBP12-YFP line (Lee et al. 2019, Nat. Comm.), however, we received seeds not containing the construct and communication with the authors of this publication indicated that there was a high propensity for the line to get silenced, and that the authors used primary transformants to produce the figures in their paper. In conclusion, we currently lack the tools to identify direct targets of UBP12 and therefore hope that our indirect way of determining the targets of UBP12 and UBP13 will suffice for the current study. To avoid confusion, we added the following line to the legend of Figure 1: "Note that above "UBP12/13 targets" refers to genes gaining H2Aub1 in ubp12/13 mutants" (lines 612-613).*

2.The proposed model is too speculative.

*a.We performed a REF6 ChIPseq experiment in ubp12/13 and included the data as figures 3D, 3E, Additional file 1: Figure S11, S12, and discussed the data at lines 237- 248. The main conclusion is that REF6 enrichment follows H2Aub1 enrichment in the ubp12/13 mutant, supporting our hypothesis of H2Aub1 being a recruiting factor for REF6.*

3.In previous study, Zhou et al. reported that H2Aub1 is widespread in the Arabidopsis genome (over 14,000 genes with this mark; Genome Biol. 18:69). It appears that only around 4,000 loci were identified with H2Aub1 in this study. Please comment on this discrepancy.

*a.One reason for this discrepancy is that we used more strict criteria to define loci marked with H2Aub1: 1) we only considered genes to be marked with H2Aub1 if they fell within the first 1 kb of genes, while Zhou et al. considered the whole gene body plus 2kb promoter. 2) we only considered peaks that overlapped in the replicates, while Zhou et al. considered also non-overlapping peaks. We analysed our H2Aub1 data with the criteria used in the other study, and found that this increased the number from 4682 to 7386. Doing the opposite, using our method on data from Zhou et al., changed the number of H2Aub1 positive from 14638 to 13019. Additionally, Zhou et al. used a single common input library instead of an input library for each extracted chromatin like in our study. This is likely to increase the false positive rate. Lastly, Zhou et al. used 7 day old seedlings, and we used 30 day old rosettes, which could explain the (major) remainder of the discrepancy. The difference in numbers of marked genes between our studies is in any case not caused by a different depth of sequencing, as in both of our H2Aub1 experiments the depth was comparable to the study of Zhou et al (4x-7x for Zhou et al., 5-8x for our study). We added a commentary about this at lines 458- 463, and Additional file 2: Table S15.*

4.Line 169 (p7): please specify the four PRC1 mutants and add a brief description on functions of relevant genes.

*a.A description has been added at lines 168-173.*

5.Lines 183-185: "This suggests that PRC1-independent H3K27me3-marked genes are already repressed before PRC2 recruitment, differing from PRC1-dependent genes that require PRC2 for repression." This sentence does not make much sense. There should be a PRC2-recruitment mechanism in all H3K27me3-marked loci.

*a.We have altered the sentence for clarity: "This suggests that PRC1-independent H3K27me3-marked genes are already repressed before PRC2 recruitment (and so Powered by Editorial Manager® and ProduXion Manager® from Aries Systems Corporation before acquiring H3K27me3), differing from PRC1-dependent genes that require PRC2 (and H3K27me3) for repression" (lines 188-190). We also altered the final sentence of the paragraph for clarification: "Together, we conclude that PRC1 likely recruits PRC2 to active genes to mediate their repression, while PRC1 appears dispensable for PRC2 recruitment to inactive genes" (lines 193-195).*

6.Lines 290-291: "Nevertheless, our study concentrated on genes gaining H2Aub1 in ubp12 ubp13 mutants, which is a consequence of UBP12/13 acting as histone deubiquitinase." Some of these genes gained H2Aub1 are expected not to be direct targets of UBP12/13.

*a.We changed the sentence to: "Nevertheless, our study concentrated on genes gaining H2Aub1 in ubp12 ubp13 mutants, which is likely a consequence of UBP12/13 acting as histone deubiquitinase" (lines 332-333).*

Reviewer #2

1.The recent study from the Calonje lab, (Gómez-Zambrano et al, 2019) should be cited and used in future revised versions of this manuscript. It has now been released publicly for several weeks and, in particular, the conclusion that PRC2 acts both in PRC1-dependent and independent ways is also presented there. Also, the fact that H2Aub ChIP-seq generated and presented here might correspond to a mix of canonical H2A and H2A.Z variants should be discussed, as both sub-populations of H2Aub could behave very differently with regard to PRC2 activity/spreading and with UBP12/UBP13 activities. Hence, it would be interesting to integrate some of the data published by Gómez-Zambrano et al in additional comparisons and analyses to determine how much of the main conclusions of the manuscript would hold true for deubiquitination of canonical H2A versus H2A.Z variant.

*a.We have included a new paragraph to address the question of whether H2A.Z (de-) ubiquitination has any bearing on our conclusions and referred to the mentioned study. We first added genome-wide H2A.Z data in ubp12/13 mutant and wt to the manuscript, as well as an analysis with data from the aforementioned study (Lines 272-290). We show that UBP12/13 targets are mildly (1.2x) but significantly enriched for H2A.Z marked genes, and also enriched for genes deregulated in hta9 HTA9-RR (a ubiquitination resistant version of HTA9) (Additional File1: Figure S14). On the other hand, UBP12/13 target genes with and without H2A.Z were similarly upregulated in ubp12/13 (Additional file1: Figure S15). This, together with the findings of GómezZambrano et al., makes it unlikely that the repressive effect mediated by UBP12/13 is connected to H2A.Z. We therefore decided not to include H2A.Z in the final model.*

2.From the bioinformatics analyses of transcriptomic and chromatin profiles in wild-type and ubp12ubp13 plants, three important mechanistic models are proposed, which unfortunately are not supported by experimental evidences at this stage. Firstly, on line 136, a list of UBP12/13 target loci is defined, which relies on a combination of transcriptomic analyses and of H2Aub profile comparisons between wild-type and ubp12ubp13 plants. Considering the importance of this statement, and the extensive usage of this list in the manuscript for many analyses, it would be important to determine actual "true" direct targets by ChIP-seq of one of these two redundant proteins.

*a.See the answer to reviewer #1, point 1.*

3.Secondly, it is concluded on line 185 that "PRC1 recruits PRC2 to active genes to mediate their repression". This conclusion makes sense but requires a real mechanistic assessment, first in term of PRC2 recruitment (for example by ChIP analysis of PRC2 proteins to PRC1-dependent loci) and by a functional assay. From the data presented, this conclusion can only presented as a working model.

*a.The sentence has been amended to: "Together, we conclude that PRC1 likely recruits PRC2 to active genes to mediate their repression, while PRC1 appears dispensable for PRC2 recruitment to inactive genes" (lines 193-195).*

4.Thirdly, the proposal that "H2Aub aids in the recruitment of the H3K27me3 demethylase REF6 needs additional support, in particular because of the great novelty of the model. For example, REF6-HA can be used for ChIP analyses on such loci in plants impaired in H2A deposition (e.g., bmi mutant plants) or H2Aub removal (ubp12/13 mutant plants).

*a.We performed a REF6 ChIPseq experiment in ubp12/13 and included the data as figures 3D, 3E, Additional file 1: Figure S11, S12, and discussed the data at lines 237- 248. The main conclusion is that REF6 enrichment follows H2Aub1 enrichment in the ubp12/13 mutant, supporting our hypothesis of H2Aub1 being a recruiting factor for Powered by Editorial Manager® and ProduXion Manager® from Aries Systems Corporation REF6.*

5.I missed the rationale for the description of decreased H2Aub levels in ubp12ubp13 mutant plants as compared to wild-type plants, as the exact opposite would be expected.

*a.We discussed this as follows: "In addition to the genes that gained H2Aub1, we unexpectedly observed twice as many that lost H2Aub1. One possible explanation is that the lack of removal of H2Aub1 from UBP12/13 targets limits the amount of available ubiquitin on other targets. Another explanation is that PRC1 function is compromised. While no gene encoding for PRC1 subunits was downregulated in ubp12 ubp13, it remains possible that UBP12/13 are required to stabilize PRC1 subunits at the protein level. The mammalian homolog of UBP12/13, USP7, deubiquitinates and thereby stabilizes a subunit of PRC1 and a similar scenario may apply to plants. This could potentially* *cause an underestimation of the number of loci on which H2Aub1 is removed, which however, should not affect the general conclusions drawn from this work" (lines 329-342).*

6.ChIP-seq data showing low H3K27me3 levels in ubp12ubp13 mutant plants should be supported by western-blots, as done for the high levels of H2Aub. In the absence of internal standards for normalization, ChIP-seq is only partially quantitative. Furthermore, this complementary western-blot would exemplify the antagonistic relationships between H3K27me3 and H2Aub levels in this mutant.

*a.As suggested, we performed a H3K27me3 Western blot in wt and ubp12/13 mutants, and added the results in Additional file 1: Figure S7. The data support that H3K27me3 levels are lower in ubp12/13 compared to wild type.*

7.I found no way to examine the ChIP-seq profiles generated in this study, as GEO accession requires a token. A token might have been generated for the reviewers but I did not found it in the data available. The anti-H2Aub antibody is notoriously difficult, and one would like to inspect the quality of the ChIP-seq presented here by looking at specific loci instead of relying on meta-gene analyses that smoothen even the dirtiest ChIP profiles.

*a.We apologize for not including the token. The data under number GSE131756 is now accessible with token: "olcfoekyjlsrded" (no quotation marks). We also added a figure showing genome browser tracks of a handful of genes (Additional file 1: Figure S17).*

8.On line 60, H2A mono-ubiquitination is indicated as H2AK121ub. However, there are multiple H2A variants and the numbering of the lysine residue targeted by PRC1 can change depending on each histone H2A isoform.

*a.We added the word "canonical" to that sentence (lines 59-61). HTA1, HTA2, HTA10, and HTA13 have a very similar amino acid sequence and the lysine in question occurs in these isoforms all on position 121.*

9.In the Methods, there is no indication on the number of biological replicates for RNAseq and ChIP-seq experiments. From the Supplementary figures, I understand 2 replicates were used in each RNA-seq or ChIP-seq experiment. Please detail this and how both replicates where integrated for all bioinformatics analyses.

*a.We apologize for the omission. We have now added the following lines to the method section: "The RNA-seq experiment was done with 3 biological replicates per genotype" (lines 394-395) and "The ChIP-seq experiments were done using 2 biological replicates per IP per genotype" (lines 417-418). We detailed in the methods how the replicates for each experiment were treated.*

10.From the Methods I understand that RNA-seq have been performed on Arabidopsis seedlings whereas ChIP-seq have been performed on rosette leaves. The rationale for this choice is surprising. This information should be stated in the main text and eventually discussed.

*a.Preferably, both types of experiments should have been performed on the same material. Because of this drawback, we have limited the number of analyses containing both our expression and our ChIP-seq data to only Figure 1c, and Additional file 1: Figure S15. We added this information in the main text here: "In order to determine whether there are UBP12- or UBP13-specific functions we performed a transcriptome analysis on both single mutants and this double mutant (two-week old seedlings)" (lines 96-98). And here: "To elucidate the mechanism by which UBP12/13 cause gene repression, we identified genes targeted by UBP12/13 by performing H2Aub1 Powered by Editorial Manager® and ProduXion Manager® from Aries Systems Corporation chromatin immunoprecipitation followed by sequencing (ChIP-seq) on 33 DAG rosette leaves of the ubp12/13 mutant and wt" (lines 123-125). We also clearly specified the material used in previously published studies that we included in our analyses by adding additional information to the figure legends: "The* *biological material underlying the RNAseq data in this figure is 2 week old whole plants (bmi1a/b, clf swn, and ubp12/13) and 3 week old whole plants (emf1). ChIP-seq data was obtained from 33 DAG rosettes" (lines 613-615). "The biological material underlying this figure is whole seedlings of 7 DAG (ChIP-seq data), 10 DAG (RNA-seq data in panels A and B*), and 14 DAG (RNA-seq data in panels B**, C, D)" (lines 629-631). "The biological material used to generate the data in this figure was 33 DAG rosettes, apart from the REF6-HA data, which was obtained from 12 DAG seedlings" (lines 653-654).*

11.The individual panels of the figures are poorly cited in the text, making the readership more difficult. Also, the numbering of Figure 3 in the text should be corrected (3D cited instead of 3B) and in the legends (C, 1552 genes and not E, 1552 genes; D, 2342 genes and not F, 2342 genes).

*a.This issue has been addressed.*

12.Related to the GEO datasets accessibility, in most instances, the definition of PRC1-dep, PRC1-indep, or other gene/peak types could be exemplified by representative browser profiles in the Figures, helping the reader to visually inspect how these different contexts look like.

*a.We added genome browser profiles in Additional file 1: Figure S17*

**Second round of review**

**Reviewer 1**

The revised version has been improved, and my concerns have been addressed satisfactorily except Point 1. I still think that a ChIP-seq analysis needs to be conducted to identify the direct targets of UBP12/13. Currently, the genes gained H2Aub1 in ubp12/13 are considered as direct targets. However, there are a large number of genes with a loss of H2Aub1 in ubp12/13 (about 4 times of the genes with H2Aub1 gain), among which there are likely direct targets of UBP12/13. The current list of direct targets of UBP12/13 is inaccurate and noninclusive.

**Reviewer 2**

The authors have addressed all the main issues I had underlined, either by adding a few new experiments (e.g., REF6 ChIP-seq in ubp12ubp13), new bioinformatics analyses, and very intensive text editing. Many supplementary figures have also been introduced, and the GEO accession number has been provided to the reviewers. Importantly, the authors also confirmed that H2Aub enrichment in ubp12ubp13 mutant plants is actually true and not a ChIP-seq normalization issue. They also addressed the links between UBP12/13 function and the H2A.Z histone variant.

It is a pity that author's attempts to identify UBP12/13 direct chromatin binding sites were unsuccessful, especially after receiving irrelevant (published) seed batches. UBP12/13 genome-wide profiles and corresponding comparisons with H3K27me3 and H2Aub profiles is an information that would ideally be needed here, but the manuscript is now cautious in the way UBP12/13 targets are called and on the general conclusions.
